# Highly‐Strong and Highly‐Tough Alginate Fibers with Photo‐Modulating Mechanical Properties

**DOI:** 10.1002/advs.202402949

**Published:** 2024-08-29

**Authors:** Lei Zhang, Qianyao Du, Jia Chen, Yun Liu, Jiahao Chang, Zhongtao Wu, Xiliang Luo

**Affiliations:** ^1^ Key Laboratory of Optic‐electric Sensing and Analytical Chemistry for Life Science MOE Shandong Key Laboratory of Biochemical Analysis College of Chemistry and Molecular Engineering Qingdao University of Science and Technology Qingdao 266042 China; ^2^ Guangdong Key Laboratory for Research and Development of Natural Drugs Guangdong Medical University Zhanjiang 524023 China; ^3^ School of Clinical Medicine Shandong Second Medical University Weifang 261053 China

**Keywords:** azobenzene, fiber, photoresponsive biomaterial, robust biomaterial, tough biomaterial

## Abstract

The good combination of high strength and high toughness is a long‐standing challenge in the design of robust biomaterials. Meanwhile, robust biomaterials hardly perform fast and significant mechanical property changes under the trigger of light at room temperature. These limit the application of biomaterials in some specific areas. Here, photoresponsive alginate fibers are fabricated by using the designed azobenzene‐containing surfactant as flexible contact point for cross‐linking polysaccharide chains of alginate, which gain high mechanics through reinforced plastic strain and photo‐modulating mechanics through isomerization of azobenzene. By transferring molecular motion into macro‐scale mechanical property changes, such alginate fibers achieve reversible photo‐modulations on the mechanics. Their breaking strength and toughness can be photo‐modulated from 732 MPa and 112 MJ m^−3^ to 299 MPa and 27 MJ m^−3^, respectively, leading to record high mechanical changes among the developed smart biomaterials. With merits of good tolerance to pH and temperature, fast response to light, and good biocompatibility, the reported fibers will be suitable for working in various application scenarios as new smart biomaterials. This study provides a new design strategy for gaining highly‐strong and highly‐tough photoresponsive biomaterials.

## Graphical abstract

Robust alginate fibers are fabricated by using the designed azobenzene‐containing surfactant as the flexible contact point for cross‐linking polysaccharide chains of alginate, which gain high mechanics through reinforced plastic strain and photo‐modulating mechanics through isomerization of azobenzene. With good tolerance to pH and temperature, fast response to light and good biocompatibility, such fibers can work in various application scenarios.

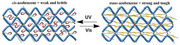

## 1 Introduction

External stimuli‐responsiveness is an attractive property for smart materials, which allows the functional materials to gain physicochemical changes in controllable ways. Mechanical force, temperature, light, electrical/magnetic field, moisture, and chemicals have been used for establishing such working systems. Among them, light usually outperforms other stimuli by gaining merits of spatiotemporal control, waste‐free and less invasion. With incorporating photochromic molecules, numerous interesting photoresponsive materials have been disclosed.^[^ ^1^, ^2^, ^3^, ^4^, ^5^, ^6^, ^7^, ^8^ ^]^ Structurally, azobenzene could perform a greater conformational change reversibly under the stimuli of two different wavelengths than other photochromic molecules like spiropyran and diarylethene. This property has flourished the development of various smart materials based on azobenzene.

Upon UV and Vis irradiations, azobenzene absorbs light energy for performing *trans‐cis* isomerization that is a process of transiting light energy to mechanical energy. The mechanical motion of azobenzene renders an ideal way for gaining optomechanics including actuation,^[^ ^9^, ^10^, ^11^, ^12^ ^]^ shape memory,^[^ ^13^ ^]^ motion,^[^ ^14^ ^]^ porosity tuning,^[^ ^15^ ^]^ and morphology manipulation.^[^ ^16^ ^]^ The photoisomerization of azobenzene can also be the driving force for the phase transition of some solid materials, advancing the fabrication of self‐healing materials,^[^ ^17^, ^18^, ^19^, ^20^, ^21^ ^]^ energy storage materials,^[^ ^22^, ^23^, ^24^ ^]^ smart adhesives^[^ ^25^, ^26^, ^27^, ^28^, ^29^, ^30^, ^31^, ^32^ ^]^ and functional biomaterials.^[^ ^33^, ^34^, ^35^ ^]^ In stark contrast, the photo‐modulation of mechanical properties, especially the breaking strength, stiffness, and toughness, of bulky materials remains a great challenge.^[^ ^36^, ^37^, ^38^, ^39^ ^]^ Furthermore, it would be more difficult for the robust materials to address fast and significant mechanical property changes under a single external stimulus of light at room temperature (r.t.). Thereby, translating molecular motion into controllable mechanical performances in macro scale becomes of great interest to researchers, which could promote the development of photoresponsive robust materials.

In the design of robust biomaterials, one more long‐standing challenge is the combination of mutually exclusive mechanical properties, for example, the strength and toughness.^[^ ^40^, ^41^, ^42^, ^43^ ^]^ Intrinsic and extrinsic mechanisms have been applied for solving this problem through functioning before and after the crack tip, respectively. As compared to the compensation of crack in the extrinsic strategy, the intrinsic way could keep the materials away from being cracked by external forces, thanks to the local energy dissipating through plastic deformation or local strain hardening near the crack tip.^[^ ^42^, ^43^, ^44^, ^45^ ^]^ For instance, interfacial hydrogen‐bond,^[^ ^46^, ^47^ ^]^ recombinant proteins,^[^ ^48^, ^49^ ^]^ inorganic nanoparticles^[^ ^50^ ^]^ and water molecules^[^ ^51^ ^]^ have been used to combine the exclusive mechanics of polysaccharide materials in the intrinsic way. While, thus far, it still lacks an intrinsic designing strategy for obtaining robust biomaterials with a fast and great photo‐modulation on both strength and toughness.

**Figure 1:**
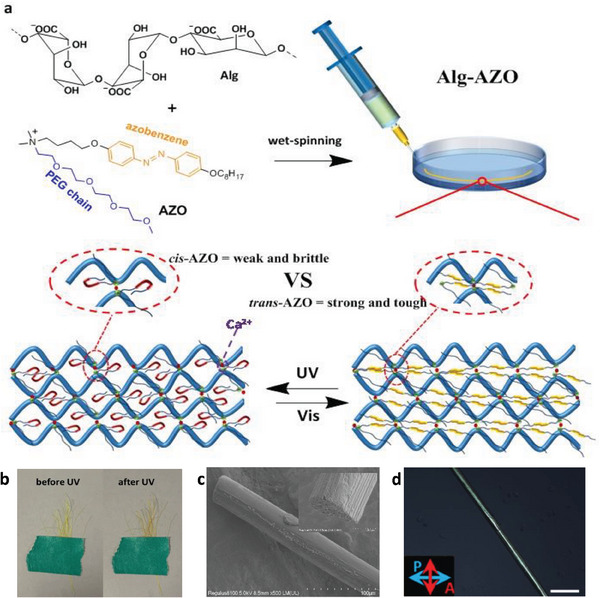
a) Schematic representation of the wet‐spinning method for fabricating photoresponsive robust Alg‐AZO fibers. b) Image of Alg‐AZO fibers before and after UV irradiation. c) SEM image of an Alg‐AZO fiber. Diameter: 43 µm. d) POM image of an Alg‐AZO fiber. Scale bar: 200 µm. All the fibers were fabricated with conditions of Alg:AZO = 20:1 and CaCl_2_% = 0.6% at r.t.

In this work, we present a design strategy for fabricating highly‐strong and highly‐tough fibers with outstanding photoresponsive mechanics. Such fibers (Alg‐AZO) are fabricated by alginate (Alg) and azobenzene‐containing surfactant (AZO) via electrostatic force (**Figure** **1**a). Structurally, the polysaccharide chain of Alg affords high strength but with poor extensibility, and the commonly used multivalent ions (Ca^2+^, Al^3+^)^[^ ^52^ ^]^ for cross‐linking polysaccharide chains also mainly contribute to strength. In order to gain tough Alg fibers, here, we use AZO molecules as flexible contact points between polysaccharide chains by taking advantage of π─π interaction among azobenzene motifs and van der Waals force among aliphatic chains. Such a non‐covalent network could enhance the binding interaction and meanwhile facilitate the energy‐dissipating slippage of polysaccharide chains, leading to Alg‐AZO fibers with high strength and high toughness. In addition, the *trans‐* and *cis‐*AZO provide different contact strengths between polysaccharide chains, endowing the fibers with the ability of responding to light. The fabricated Alg‐AZO fibers can reach maximum breaking strength of 732 MPa and toughness of 112 MJ m^−3^, while, upon a short time of UV treatment, these mechanics would be decreased to 299 MPa and 27 MJ m^−3^, respectively. More interestingly, such fibers exhibit good biocompatibility, wide temperature and pH tolerances, suitable for working at various harsh conditions.

## 2 Results and Discussion

### 2.1 Synthesis and Characterizations of Alg‐AZO Fibers

As two major components for the synthesis of Alg‐AZO fibers, Alg was purchased and used directly without further purification, and AZO was synthesized as an orange solid over five steps in 34% yield (Schemes S1 and S2, Supporting Information). Besides, Ca^2+^ was also used for cross‐linking polysaccharide chains of Alg to improve the strength of fibers. AZO is a quaternary ammonium compound with an azobenzene‐containing chain and a polyethylene glycol (PEG) chain. As the contact points for cross‐linking polysaccharide chains, AZO molecules would form π─π interaction and van der Waals force between the azobenzene‐containing chains. Although these are weaker forces than covalent connection and electrostatic interaction, the accumulation of such forces would enhance the cross‐linking of polysaccharide chains together with the interactions from H─bond^[^ ^48^ ^]^ and Ca^2+^, which improve both breaking strength and toughness of Alg‐AZO fibers. The presence of PEG chain, on one hand, increases the water solubility of AZO for facilitating its ionic complexation with Alg in aqueous condition, and on the other hand, can reinforce the binding of AZO to Alg chain by forming non‐covalent interactions.

At r.t., the first diffraction peak at 0.17 Å^−1^ and following harmonic peaks in small angle X‐ray scattering (SAXS) profile indicated a smectic ordered structure of AZO (Figure S1, Supporting Information). And the ordered structure could be confirmed by polarized optical microscopy (POM) analysis (Figure S2, Supporting Information). To assess the photoswitching ability, AZO was first irradiated by UV light in aqueous solution at r.t. (Figure S3, Supporting Information), showing typical *trans‐to‐cis* isomerization characteristics of π─π^*^ peak decrease at 360 nm and n‐π^*^ peak increase at 450 nm. Vis light restored π─π^*^ and n‐π^*^ peaks back by inducing the *cis‐to‐trans* isomerization. In solid state, AZO could also partially isomerize under UV irradiation thanks to the flexible structure from double chains, evidenced by a smaller degree of UV–vis absorption change than that in solution (Figure S4, Supporting Information). The thermodynamic stability of *cis*‐isomer is crucial for maintaining the UV‐induced properties of azobenzene‐containing materials. Upon recording the UV–vis absorption changes in dark at r.t., half‐lives of *cis‐*AZO in aqueous solution and solid state were obtained as 18.0 and 14.4 h, respectively (Figures S5 and S6, Supporting Information). The reduced half‐life of solid‐state *cis‐*AZO is presumably caused by the strong intermolecular interactions in tightly packed solid that energetically favor the formation of planar *trans*‐AZO.^[^ ^43^ ^]^ The *cis*‐AZO% in photostationary state (PSS) were determined to be 94% and 18% in solution and solid state by ^1^H NMR analyses, respectively (Figures S7 and S8, Supporting Information).

The Alg‐AZO fibers from Alg, AZO, and Ca^2+^ were produced by applying a wet‐spinning methodology (Figure 1a). First, the aqueous mixture of Alg and AZO was injected into a solution of CaCl_2_ in an “S” shape, and the resulted fibers were left in CaCl_2_ solution for 5 min for a sufficient cross‐linking between polysaccharide chains and cationic species of AZO + Ca^2+^ before the collection by a rotating cylinder. Then the obtained fibers were post‐stretched to further enhance their mechanics.^[^ ^53^, ^54^, ^55^ ^]^ Finally, the stretched fibers were dried for 20 min in an ambient condition (r.t., relative humidity (RH) < 30%) to afford the photoresponsive fibers of Alg‐AZO (Figure 1b). The successful ionic complexation between Alg and AZO could be confirmed by the color change from light yellow to dark yellow under UV (Figure 1b) and FT‐IR characterizations (Figure S9, Supporting Information). According to the scanning electron microscopy (SEM) analysis, the as‐prepared Alg‐AZO fibers were in a uniform size, and the cross‐section analysis showed a solid internal core (Figure 1c). The bright birefringence in POM analysis indicated the ordered structure of Alg‐AZO (Figure 1d). The thermogravimetric analysis (TGA) revealed that the as‐prepared Alg‐AZO fibers contained water less than 8% and gained thermal integrity higher than 200 °C (Figure S10, Supporting Information).

### 2.2 Mechanical Properties of Alg‐AZO Fibers

**Figure 2:**
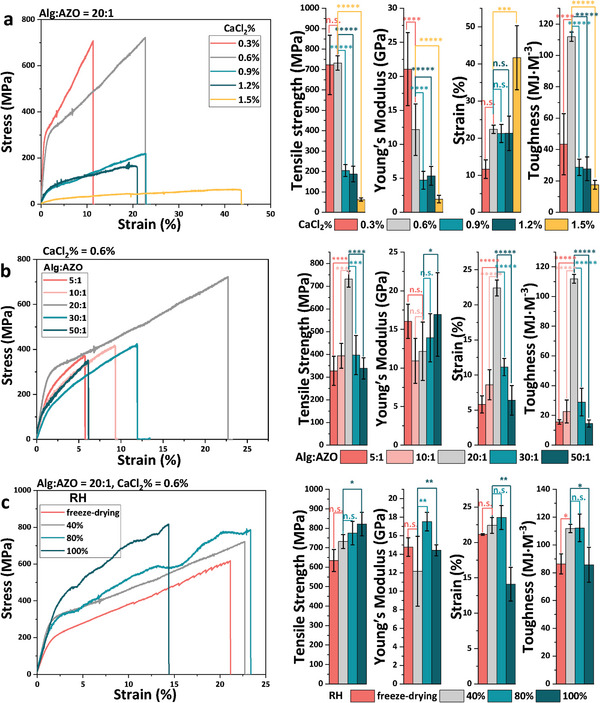
Mechanical properties of Alg‐AZO fibers. a) Fibers extruded from CaCl_2_ solutions with mass concentrations from 0.3% to 1.5%. Alg:AZO = 20:1. b) Fibers with different charge ratios of Alg:AZO from 5:1 to 50:1. CaCl_2_% = 0.6%. c) Fibers incubated under different RHs from freeze‐drying to 100 °C. Alg:AZO = 20: 1, CaCl_2_% = 0.6%. Diameters: 20–40 µm. Statistical significance: n.s. = not significant, ^*^ *p* < 0.05, ^**^ *p* < 0.01, and ^***^ *p* < 0.001, ^****^ *p* < 0.0001, ^*****^ *p* < 0.00001.

Subsequently, the mechanical properties of Alg‐AZO fibers were evaluated by a tensile testing machine. For gaining robust Alg‐AZO fibers, the mass concentration of CaCl_2_ solution, stoichiometric charge ratio of Alg:AZO and water content of fibers were screened. As one of the widely used ions in the fabrication of Alg‐based fibers, Ca^2+^ can coordinate with the COO^−^ groups of Alg to form a cross‐linking network,^[^ ^56^, ^57^ ^]^ which effectively improves the mechanical properties of fibers, especially for the breaking strength. With a mixed solution of Alg:AZO = 20:1, the extruded fibers from 0.3%, 0.6%, 0.9%,1.2% and 1.5% CaCl_2_ solutions were prepared and compared (Figure S11 and Table S1, Supporting Information). Along with the CaCl_2_% increasing from 0.6% to 1.5%, a decreasing trend of breaking strength and Young's modulus was observed (**Figure** **2**a), which should be caused by the concentration change of Ca^2+^‐based “egg‐box” sites between polysaccharide chains. An investigation of the impacting effect of CaCl_2_% on the mechanical properties showed that the amount of Ca^2+^ should be carefully controlled for gaining strong Alg‐based fibers as two much “egg‐box” sites disfavor the cross‐linking between polysaccharide chains.^[^ ^56^ ^]^ It is worth noting that a lower concentration of CaCl_2_ was optimized in our work than those reported values, which should be caused by the joint use of AZO as additional contact points. Compromisingly, with 0.6% CaCl_2_ solution the obtained Alg‐AZO fibers showed both high breaking strength (732 MPa) and high toughness (112 MJ m^−3^).

With the optimized CaCl_2_ concentration, the influence of Alg:AZO on the mechanics was investigated by changing the ratio from 5:1 to 50:1 (Figure S12 and Table S2, Supporting Information). Alg‐AZO performed low breaking strength and extensibility with using too much or less amount of AZO (ratio = 5:1, 30:1 and 50:1) (Figure 2b). By adjusting AZO:Alg to be 20:1, maximum breaking strength, strain, and toughness would be obtained. Interestingly, the Young's modulus of Alg‐AZO was not very sensitive to the content change of AZO, due to the fact that the presence of AZO mainly contributes to the plastic strain of fibers, vide infra. The water content is another key factor that impacts the mechanical performances of polysaccharide‐based fibers,^[^ ^48^, ^51^ ^]^ which might be changed upon the actual environmental humidity condition. Alg‐AZO fibers were incubated in different RH conditions at r.t. for checking the humidity impact on the mechanics (Figure S13 and Table S3, Supporting Information). It turned out that Alg‐AZO exhibited almost consistent mechanical properties under different RH conditions (Figure 2c), which revealed the hydrophobic nature of such fibers. Here, the absence of the hydrophilic property of PEG chain would be probably caused by its strong interaction with polysaccharide chain, vide infra. Thereby, in this work, CaCl_2_% = 0.6%, Alg:AZO = 20:1 and a 20 min drying process at low RH conditions at r.t. were used as the optimal condition for fabricating robust Alg‐AZO fibers. With such conditions, the fabricated fibers gained a good reproducibility of mechanical performances (Figure S12f, Supporting Information).

### 2.3 Working Mechanism of AZO Molecules as Flexible Contact Points

**Figure 3:**
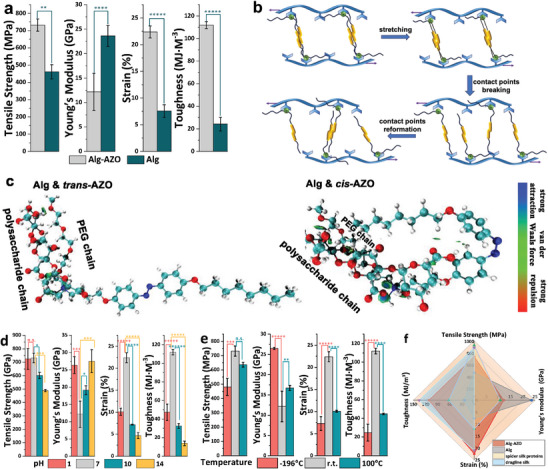
a) Mechanical properties of Alg‐AZO and Alg fibers. b) Contribution of AZO to the high mechanical performances of Alg‐AZO fibers through dynamic breakage and reformation of contact points. c) Computational calculation on the interactions between *trans‐*/*cis‐*AZO and polysaccharide chain. Mechanical properties of Alg‐AZO fibers treated by different d) pH and e) temperatures. f) Spider radar for comparing the mechanical performances of Alg‐AZO fibers, Alg fibers, spider silk proteins, and dragline silks. Diameters: 20∼40 µm. Statistical significance: n.s. = not significant, ^*^ *p* < 0.05, ^**^ *p* < 0.01, and ^***^ *p* < 0.001, ^****^ *p* < 0.0001, ^*****^ *p* < 0.00001.

In the design of Alg‐AZO, AZO molecules work as the flexible contact points between polysaccharide chains by forming π─π interaction from azobenzene motifs and van der Waals forces from linear chains, differentiating from the rigid cross‐linking points of Ca^2+^. Ca^2+^ and AZO would mainly contribute to the elastic deformation and plastic deformation of Alg‐AZO fibers, respectively. To prove this fact, pure Alg fibers were prepared by following a similar synthetic procedure as that of Alg‐AZO but without using AZO, which gave a stress–strain profile with much reduced plastic region (Figure S14 and Table S4, Supporting Information). The lack of flexible contact points largely limited the breaking strength, extensibility, and toughness of Alg fibers, but afforded higher elastic modulus than Alg‐AZO (**Figure** **3**a). Working as the joint contact points with Ca^2+^, AZO molecules could promote the formation of a hybrid cohesive matrix between polysaccharide chains,^[^ ^48^ ^]^ efficiently interlocking sugar chains but still allowing sufficient energy‐dissipating slippage. During the stretching process, the π─π interaction and van der Waals force between AZO molecules could be dynamically broken and reformed (Figure 3b). This would permit a large distance of interfacial slipping between polysaccharide chains, as compared to the covalent bond connection for cross‐linking polysaccharide chains. In this way both strength and toughness are enhanced, leading to Alg‐AZO fibers with highly‐strong and highly‐tough properties. However, in the fabrication of such fibers, the amount of AZO should be carefully controlled (Figure 2b). With a large content of AZO, the abundant contact points would inhibit the energy‐dissipating sliding of sugar chains, and a wide distribution of AZO with phenyl rings would also destroy the contacting points of H─bonds between sugar chains. In contrast, the less use of AZO would bring insufficient energy‐dissipating slippage when stretching the fibers. All of these weaken the mechanics of Alg‐AZO fibers.

Additionally, we found the presence of PEG chain could improve the binding interaction between AZO and polysaccharide chain. Computational calculations on the intermolecular interactions between polysaccharide chain and *trans‐*/*cis‐*AZO were performed by using density functional theory (DFT) B3LYP functional^[^ ^58^ ^]^ coupled with Grimme D3 dispersion correction^[^ ^59^ ^]^ and 6–31G(d,P) basis set and analyzed by independent gradient model based on Hirshfeld partition of molecular density (IGMH) method.^[^ ^60^, ^61^ ^]^ Meanwhile, an assumption that there would be H─bond interaction between −OH group of polysaccharide chain and oxygen atom of PEG was applied in the modeling process, with considering the hydrophilic property of PEG chain and the hydrophobic property of azobenzene‐containing chain. The *sign(λ_2_)ρ* mapped δ_g_^inter^ isosurfaces between polysaccharide chain and PEG chain present blue and green isosurfaces (Figure 3c), indicating that the H─bond and van der Waals force are the major intermolecular interactions. As a quantitative descriptor of inter‐fragment interaction, *δ*_g_^inter^ indices between Alg and *trans‐*/*cis‐*AZO were also calculated, providing 0.76 a.u. for Alg & *trans‐*AZO and 1.18 a.u. for Alg & *cis‐*AZO, which confirmed the strong interaction between polysaccharide chain and PEG chain. Interestingly, *cis‐*AZO showed a stronger binding interaction with polysaccharide chain than *trans*‐AZO, which would be probably caused by their different existing states. Planar *trans‐*AZO molecules prefer to stack together through π─π interaction and van der Waals force between *trans‐*AZO molecules. Such a stacking state would lead to each *trans*‐AZO molecule suffering intermolecular interactions from both “head” direction and “tail” direction. In contrast, non‐planar *cis*‐AZO molecules could not stack together, leading to each *cis*‐AZO molecule suffering intermolecular interactions only from the “head” direction, which results in their stronger binding interaction to Alg than that of *trans*‐AZO molecules to Alg. These calculations confirmed that the *trans‐to‐cis* isomerization of AZO would not weaken the binding interaction to Alg. Thereby, the weakened mechanics induced by UV light should be solely caused by the intermolecular interaction changes between AZO molecules, vide infra.

### 2.4 Tolerance of Alg‐AZO Fibers to Extreme Conditions

The tolerance to harsh conditions determines the application scenarios of functional materials. Here, to test the pH tolerance, Alg‐AZO fibers were treated by aqueous solutions with pH 1–14 for 5 min and then dried for tensile tests (Figure S15 and Table S5, Supporting Information). As is shown in Figure 3d, the pH 1 condition largely reduced the extensibility and toughness of Alg‐AZO fibers and meanwhile stiffened the fibers, which should be caused by the reduction of flexible contact points of AZO in the fibers. In pH 1 condition, the rich H^+^ could partially replace the cationic AZO and Ca^2+^ and then promote the formation of new contact points of H─bonds between polysaccharide chains. As a result, the lack of flexible contact points limited the energy‐dissipating slippage of polysaccharide chains, thereby reducing the extensibility and toughness of fibers. However, even treated by pH 1 condition, Alg‐AZO fibers exhibited better mechanical performances than pure Alg fibers (Figure S14b, Supporting Information), thanks to the contribution of remaining AZO molecules after ion exchanges. The UV–vis absorption measurements clearly indicated the presence of AZO in pH 1 treated fibers, albeit with a lower concentration than as‐prepared fibers (Figure S16, Supporting Information). The strong interactions between PEG chain and polysaccharide chain contributed to the remaining of AZO molecules under acidic conditions. In contrast, in basic conditions with pH >10, the high concentration of OH^−^ would neutralize the cationic Ca^2+^ and AZO and break the interactions (mainly H─bonds) between PEG chain and polysaccharide chain, which dramatically weakened the mechanics of Alg‐AZO fibers to a level of pure Alg fibers. The low concentration/absence of AZO in pH 10/14‐treated fibers were also confirmed by UV–vis absorption studies (Figure S16, Supporting Information).

In the following, the temperature tolerance of Alg‐AZO fibers was investigated (Figure 3e; Figure S17 and Table S6, Supporting Information). Both extreme cold conditions (liquid nitrogen) and heating conditions (100 °C) stiffened the fibers and reduced the strength, strain, and toughness, which should be caused by the physical state change and content change of water. The as‐prepared Alg‐AZO fibers contained ≈8% water (Figure S10, Supporting Information). Treated by liquid nitrogen, the free water would be converted to be ice clusters and then lead to internal defects in the matrix of Alg‐AZO, while, under heating conditions free water would be evaporated and then hamper the energy‐dissipating slippage of polysaccharide chains. Thereby, either extreme cold conditions or heating conditions could stiffen the fibers. However, of note, Alg‐AZO fibers maintained impressive breaking strength in all the tests ranging from pH 1–14 and −196–100 °C.

By applying AZO as joint points with Ca^2+^, highly‐strong and highly‐tough alginate fibers were successfully fabricated, with showing attractive mechanics including breaking strength of 732 MPa, strain of 22%, and toughness of 112 MJ m^−3^. Such fibers well combine the mutually exclusive properties of strength and toughness together. The spider radar chart clearly indicates that Alg‐AZO fibers address high mechanics close to some of the natural dragline silks (Figure 3f), outperforming most of the artificial materials (**Table** **1**), especially in gaining high toughness for highly‐strong materials.

**Table 1:** Mechanical properties of Alg‐AZO fibers and other reported robust materials.

Entry	Materials	Tensile strength [MPa]	Young's modulus [GPa]	Strain [%]	Toughness [MJ m^−3^]
1	Alg‐AZO in this work	731.80 ± 35.50	12.16 ± 3.78	22.40 ± 1.13	111.88 ± 2.96
2	Alg in this work	461.65 ± 41.26	23.63 ± 2.14	7.58 ± 1.18	24.20 ± 6.02
3	WS‐PSD‐3x^[^ ^62^ ^]^	505		44	
4	spider silk proteins^[^ ^63^ ^]^	1030 ± 110	13.7 ± 3.0	18±6	114 ± 51
5	CNF/silk^[^ ^64^ ^]^	1015	55	10	55
6	CNF/K144^[^ ^64^ ^]^	466.7 ± 58.2	21.3 ± 3.0	10.8 ± 1.0	34.0 ± 3.1
7	Alg/RE‐stretch^[^ ^65^ ^]^	768	24		69
8	dragline silk^[^ ^66^ ^]^	880–1500		21–27	136–194
9	NaAlg/GO^[^ ^67^ ^]^	620	4.3		
10	Alginate filaments^[^ ^68^ ^]^	500	28		
11	GHB fibers^[^ ^69^ ^]^	129.8	5–5.6	13	
12	BC^[^ ^70^ ^]^	826	65.7		
13	hair^[^ ^71^ ^]^	150–270			
14	high‐tensile steel^[^ ^66^ ^]^	1500		0.8	6

### 2.5 Photo‐Modulation on the Mechanical Properties of Alg‐AZO Fibers

**Figure 4:**
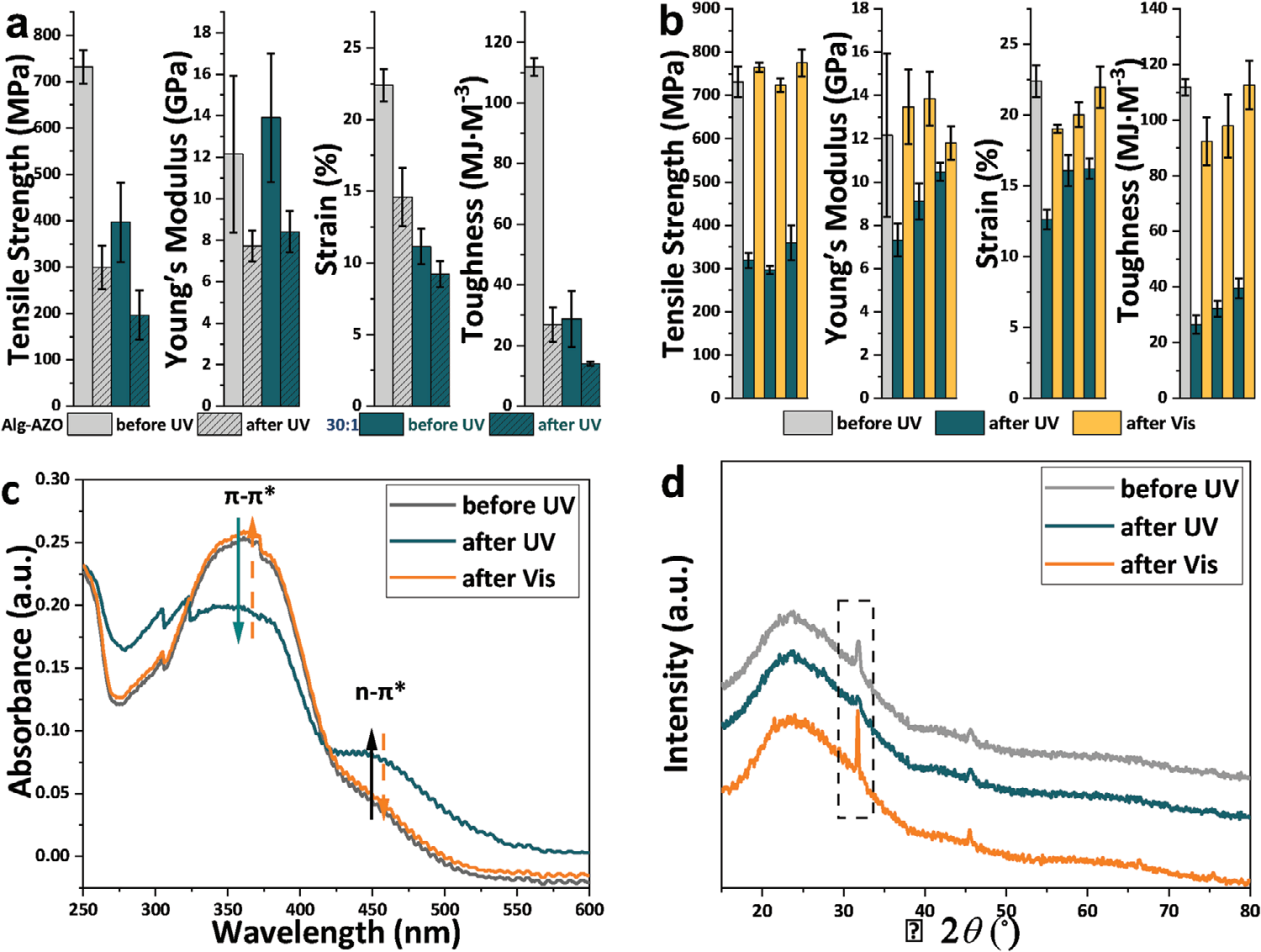
a) Mechanical property changes of Alg‐AZO and Alg‐AZO (30:1) fibers before and after UV irradiation at r.t. b) Mechanical property changes of Alg‐AZO fibers under switching UV–vis irradiations at r.t. Diameters: 20–40 µm. c) UV–vis absorption and d) XRD changes of Alg‐AZO fibers before and after UV/Vis irradiation at r.t. The samples used for these two tests were only irradiated by lights during the measurements.

In Alg‐AZO fibesr, AZO molecules are the contact points for cross‐linking polysaccharide chains through π─π interaction among azobenzene motifs and van der Waals force among aliphatic chains. Therefore, the conformational change of azobenzene would bring the intermolecular interaction change between AZO molecules, and then impact the cross‐linking of polysaccharide chains. Specifically, planar *trans‐*AZO molecules could form an ordered molecular stacking, which works as the contact points for providing high mechanics. With UV treatment, *trans*‐AZO would isomerize to non‐planar *cis*‐AZO. The formation of *cis‐*AZO would destroy the molecular stacking and weaken the cohesive matrix between polysaccharide chains, leading to low mechanics. Based on this theory, the mechanical properties of Alg‐AZO fibers could be modulated by light at r.t. To study the photo‐modulation efficiency on mechanics, Alg‐AZO fibers were UV irradiated for 5 min at r.t. before carrying out tensile tests (Figure S18 and Table S7 – Entry 1, Supporting Information). After UV treatment, the breaking strength and toughness of Alg‐AZO fibers were reduced to 299 MPa and 27 MJ m^−3^, which are 41% and 24% of the original values (**Figure** **4**a). Of note, the conformational change of AZO mainly influenced the plastic deformation of Alg‐AZO fibers due to the role of AZO working as flexible contact point. As a result, relatively small changes of Young's modulus and strain were obtained by reaching 7.7 GPa and 14.6% which are 64% and 65% of the original values. To further confirm the photo‐modulating ability of AZO on the mechanics, Alg‐AZO (30:1) fibers were also tested, which exhibited UV‐induced mechanical property changes as well (Figure 4a; Figure S18b, Table S7 – Entry 2,Supporting Information). With a lower content of AZO, Alg‐AZO (30:1) fibers generally showed lower mechanical property changes than those of Alg‐AZO fibers. These phenomena indicated that AZO plays an important role to the mechanical properties of such fibers. Vis light is an effective stimulus for recovering the mechanics back by inducing *cis‐to‐trans* isomerization of AZO. Under switching UV–vis irradiations, breaking strength, Young's modulus, strain, and toughness of Alg‐AZO fibers showed very good cyclic changes in three repeating tests (Figure 4b; Figure S19 and Table S8, Supporting Information).

To gain more insights on the photo‐modulating mechanics, UV–vis absorption and XRD changes of Alg‐AZO fibers were recorded before and after UV/Vis irradiation. As is shown in Figure 4c, UV irradiation would decrease π─π^*^ absorption peak of azobenzene at 360 nm and increase n‐π^*^ peak at 450 nm, and Vis light could restore these peaks back to original states, clearly indicating the reversible *trans‐cis* isomerization of AZO in fibers. This proved the fact that the above mechanical property changes were caused by the isomerization of AZO. In the XRD profiles, a diffraction peak at 32°was diminished by UV and recovered by Vis (Figure 4d), which implied the ordered structure changes of Alg‐AZO fibers. In Alg‐AZO fibers, only AZO contains a photochromic motif of azobenzene that could respond to UV and Vis light by performing *trans‐cis* isomerization. As illustrated in Figure 1a, *trans*‐AZO molecules could associate polysaccharide chains via π─π interaction among azobenzene motifs and van der Waals force among aliphatic chains, thanks to the planar conformation of *trans‐*azobenzene. In such a way, *trans*‐AZO molecules form an ordered structure between polysaccharide chains, which leads to the appearance of diffraction peak at 32°. After UV treatment, the resulted non‐planar *cis*‐AZO molecules could not stack together, leading to the disordered arrangement of AZO molecules, which then causes the diminish of diffraction peak at 32°. The ordered structure is of great necessity to maintain high mechanics,^[^ ^51^, ^64^, ^70^ ^]^ and thereby the mechanical properties of Alg‐AZO fibers could be photo‐modulated.

### 2.6 Application Tests with Alg‐AZO Fibers

**Figure 5:**
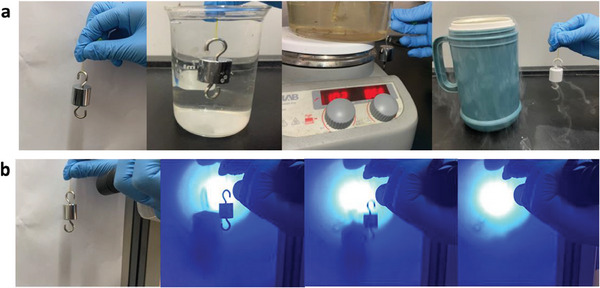
Images of Alg‐AZO fibers lifting one 50 g weight in the air, in acidic aqueous solution (pH 1), at 100 °C and in liquid nitrogen. b) Photoinduced fracture of Alg‐AZO fibers lifting one 50 g weight at r.t.

The high mechanical properties support wide applications of Alg‐AZO fibers in different environments. As is shown in **Figure** **5**a, five thin fibers (20 µm diameter for each) can lift one 50 g weight in air, in acidic aqueous solution (pH 1), at 100 °C and in liquid nitrogen. A spatiotemporal control on the mechanical property of Alg‐AZO fibers became available at r.t. by taking advantage of the photoresponsive mechanical property changes of such fibers. Also with lifting one 50 g weight, a remote UV irradiation could fracture the Alg‐AZO fiber in a short time and let the weight fall off (Figure 5b; Video S1, Supporting Information). Such properties would allow Alg‐AZO fibers to be applicable in some inaccessible environments, for example, releasing a target in the closed container, extreme acidic solution, or extreme cold condition.

It is worth noting that Alg‐AZO would be the first robust fiber material that could perform reversible photo‐modulation on the mechanics of strength, Young's modulus, extensibility and toughness in large degrees. As compared to the previously reported photoresponsive biomaterials, Alg‐AZO fibers gained much higher mechanics including over 3 times of breaking strength (732 Vs 215 MPa), 2 times of Young's modulus (12 Vs 6.8 GPa), 3 times of extensibility (22% Vs 6.5%) and 12 times of toughness (112 Vs 9.2 MJ m^−3^), and also the more powerful photo‐modulation on breaking strength (433 Vs 111 MPa), Young's modulus (4.3 Vs 3.3 GPa), extensibility (7.8% Vs invalid value) and toughness (85 Vs 2.8 MJ/m^3^).^[^ ^38^ ^]^ As is known, transferring the molecular motion into mechanical property change is remaining a great challenge for bulky materials, while, which would be more difficult for robust materials. More importantly, here we realized UV‐induced decrease and Vis‐induced increase on mechanics, which is the first example of achieving reversible modulation on the mechanics of robust biomaterials with using light as the only trigger. Furthermore, Alg‐AZO fibers also addressed a good combination of strength and toughness, being a photoresponsive strong, and tough material.

### 2.7 Biological Studies of Alg‐AZO Fibers

**Figure 6:**
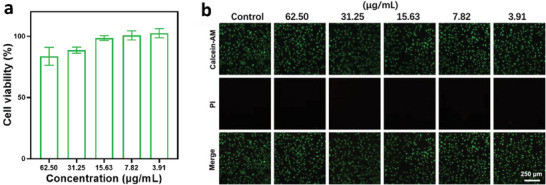
a) Cell viability of L929 cells after being treated with aqueous suspensions of Alg‐AZO fibers at various mass contents. b) Live/dead fluorescence staining images of L929 cells after being treated with Alg‐AZO. Red fluorescence and green fluorescence represent dead cells and live cells, respectively. (Calcein‐AM Ex/Em: 491/517 nm, PI Ex/Em: 535/617 nm).

In biomedical area, an ideal surgical suture should simultaneously have high strength and high toughness. For example, in the skin stitching, a strong but less stretchable surgical suture would bring a risk of cutting skin and thereby cause secondary injury on the wound position during the daily movement of muscle tissues. This risk could be well avoided by using highly‐tough sutures that could be stretched upon the movement of skin. Alg‐AZO fibers gain both high strength and high toughness, meeting the requirements of being high performance sutures. Meanwhile, the photo‐modulating property would be expected to provide a facile way for the surgical suture removal that could be achieved by cutting off the Alg‐AZO fibers through a short time of UV irradiation. This way would bring conveniences and save cost for the surgical operation by avoiding the use of disposable blades. Considering the potential application scenarios in biological and biomedical areas, the biosafety of Alg‐AZO fibers was investigated. The cell toxicity of Alg‐AZO fibers was evaluated by L929 cells in CCK‐8 assays.^[^ ^72^ ^]^ In brief, cell viabilities were obtained by incubating L929 cells with aqueous suspensions of Alg‐AZO fibers at various mass contents for 24 h at 37 °C. As shown in **Figure** **6**a, even with a high content (62.50 µg mL^−1^), Alg‐AZO could still give cell viability higher than 87% after 24 h incubation, and the in vitro toxicity would be negligible at concentrations < 31.25 µg/mL. These results indicated the very good in vitro biocompatibility of Alg‐AZO fibers. The recorded fluorescence staining images of L929 cells in this study further confirmed the biosafety of Alg‐AZO fibers by showing negligible cell death when the mass content of Alg‐AZO lower than 62.50 µg mL^−1^ (Figure 6b). Based on the good biocompatibility, Alg‐AZO fibers could be a potential biological and biomedical material with photocontrollable mechanical properties in the future applications.

## 3 Conclusion

In this study, an azobenzene‐containing surfactant AZO is designed as the contact point for cross‐linking polysaccharide chains of Alg, which is used for the fabrication of photoresponsive robust fibers. With the optimized synthetic condition, the prepared Alg‐AZO fibers combine the mutually exclusive mechanical properties by showing breaking strength of 732 MPa and toughness 112 MJ m^−3^. Alg‐AZO fibers could accommodate various application scenarios thanks to their good tolerance to harsh conditions. Based on the isomerization of AZO, the mechanics of Alg‐AZO fibers could be photo‐modulated in large degrees, by showing changes of breaking strength, Young's modulus, extensibility, and toughness from 732, 12, 22%, 112 to 299 MPa, 7.7 GPa, 14.6%, 27 MJ m^−3^, respectively. The good biocompatibility also opens an opportunity of Alg‐AZO fibers to be applicable in biological and biomedical areas, for example, being as a spatiotemporal controlled suture in surgeries. This study provides a new designing strategy with intrinsic mechanism for gaining highly‐strong and highly‐tough photoresponsive biomaterials.

## 4 Experimental Section

### Characterizations

NMR spectra were recorded on a Bruker Avance 500 machine with the solvent of CDCl_3_. POM was performed on a Nikon ECLIPSE LV100NPOL machine. Rigaku Corporation's D/MAX/2500 PC X‐ray Diffraction was used for the XRD measurements. A conventional X‐ray source was employed for SAXS measurement with the radiation wavelength of *λ* = 1.54 Å. The distance of sample‐to‐detector was set as 20 cm. *q* = 4π sinθ/λ was used for the calculation of the scattering vector, and 2θ was defined as the scattering angle. A Netzsch STA 449C thermal analyzer was used for TGA measurement with a heating and cooling rate of 10 °C min^−1^ under N_2_ atmosphere. Scanning electron microscopy (SEM) images were recorded from a JSM‐7500F field emission scanning electron microscope equipped with an energy dispersive x‐ray spectroscopy (EDS) detector. pH values were determined by a PHS‐3E pH Fmeter. The lights of UV (365 nm, 128 mW cm^−2^) and Vis (520 nm, 53 mW cm^−2^) were used for inducing the isomerization of AZO. All the irradiations were performed within 20 cm.

### Tensile Tests

Mechanical properties were tested with a Universal Testing Machine (INSTRON). All the mechanical properties were obtained by the tensile stretching tests with a 1 mm min^−1^ stretching speed. The stress–strain curves were recorded until the occurrence of fiber crack. Repeating tests were carried out for each sample. Tensile stress was calculated as the force divided by the cross‐sectional area assumed to be circular. The cross‐sectional area was calculated by using the diameter of the fiber before the test.

### Fabrication of Alg‐AZO Fibers

In this work, Alg with a chemical formula of (C_6_H_7_N_a_O_6_)_n_ was used for the fabrication of Alg‐AZO fibers through an electrostatic complexation with AZO in aqueous solution. Based on an assumption of one negative charge per monosaccharide unit, a solution mixture of Alg and AZO with stoichiometric charge ratio in 20:1 could be prepared by adding the aqueous solution of AZO (1.2 mL, 9.6 mm) to an aqueous solution of Alg (7.5 mL, 30.0 mm). Alg‐AZO fibers were extruded by injecting the solution mixture of Alg and AZO into a CaCl_2_ solution (0.6%, 50 mL) through a needle with an inside diameter of 0.5 mm. During the injection, the needle tip was below the surface of CaCl_2_ solution, and the needle tip moved in an “S” shape. A constant extrusion rate (0.2 mL S^−1^) was controlled manually. After the injection, the fibers were left in the CaCl_2_ solution for 5 min for a better cross‐linking before the collection by a rotator. The collected fibers were post‐stretched by 1.1 times of original length at a speed of 1.0 mm min^−1^ with using a tensile testing machine. After drying at ambient condition (≈20 **°**C, RH < 50%) for 20 min, Alg‐AZO fibers were obtained.

### Statistical Analysis

All the quantitative data were presented as mean ± standard deviation. All variables were checked for normality. The experimental data were processed using an origin software. Statistical significance was determined using a one‐way ANOVA followed by the Tukey Test: n.s. = not significant, ^*^ *p* < 0.05, ^**^ *p* < 0.01, ^***^ *p* < 0.001, ^****^ *p* < 0.0001, and ^*****^ *p* < 0.00001.

## Conflict of Interest

The authors declare no conflict of interest.

## Supporting information

Supporting Information

Supplemental Video 1

## Data Availability

The data that support the findings of this study are available in the supplementary material of this article.
