# Supplementary material for: Highly‐Strong and Highly‐Tough Alginate Fibers with Photo‐Modulating Mechanical Properties
Source: Adv Sci (Weinh). 2024 Aug 29;11(40):2402949. doi: 10.1002/advs.202402949 (PMC11516064; doi:10.1002/advs.202402949)
Supplement: Supplementary file 1 — Supporting Information [file ADVS-11-2402949-s001.pdf]

ADVANCED SCIENCE
Open Access

Supporting Information

for *Adv. Sci.*, DOI 10.1002/advs.202402949

Highly-Strong and Highly-Tough Alginate Fibers with Photo-Modulating Mechanical Properties

Lei Zhang, Qianyao Du, Jia Chen, Yun Liu, Jiahao Chang, Zhongtao Wu and Xiliang Luo**

Supporting Information

Highly-strong and Highly-tough Alginate Fibers with Photo-modulating Mechanical Properties

Lei Zhang, Qianyao Du, Jia Chen, Yun Liu, Jiahao Chang, Zhongtao Wu, Xiliang Luo**

1. General remarks

2. Synthesis and characterizations of AZO

3. Fabrication of Alg-AZO and Alg fibers

4. Characterizations of Alg-AZO and Alg fibers

5. Intermolecular interaction analyses by computational calculation

6. Biological studies of Alg-AZO fibers

1. General remarks

All the reagents and solvents for synthesizing AZO, including 4-acetylaminophenol, K_2CO_3, 1-bromooctane, 1,8-dibromooctane, sodium hydroxide, sodium nitrite, phenol, dichlorosulfoxide, acetonitrile, acetone, petroleum ether, ethyl acetate, methylene chloride, methanol, acetonitrile, 35% hydrochloric acid were purchased and used directly. Sodium alginate and $CaCl_2$ were purchased and used directly. Cell Counting Kit-8 (CCK-8) was purchased from Shanghai Beyotime Biotechnology Co, Ltd. (Shanghai, China). Dulbecco's modified Eagle's medium (DMEM) and fetal bovine serum (FBS) were purchased from Gibco. Propidium iodide (PI) and Calcein acetoxymethyl ester (Calcein-AM) were purchased from Shanghai Beyotime Biotechnology Co, Ltd. (Shanghai, China).

2. Synthesis and characterizations of AZO

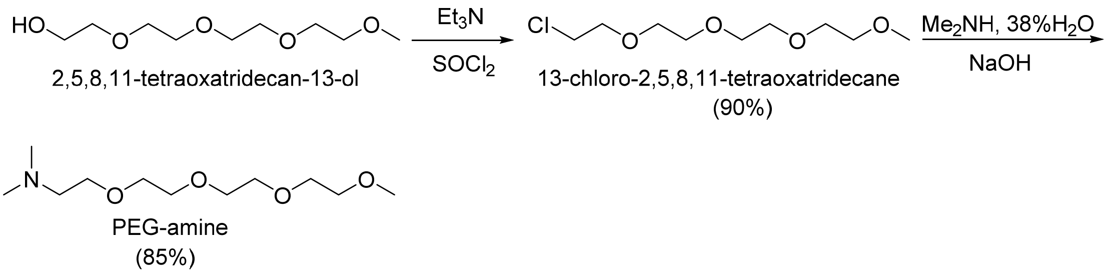

Scheme S1. Synthesis of PEG-amine.

***N,N*-dimethyl-2,5,8,11-tetraoxatridecan-13-amine (PEG-amine):** PEG-amine was synthesized as a dark-brown oil by following the reported procedures.[1] ^1H NMR is in consistence with that of reported data.

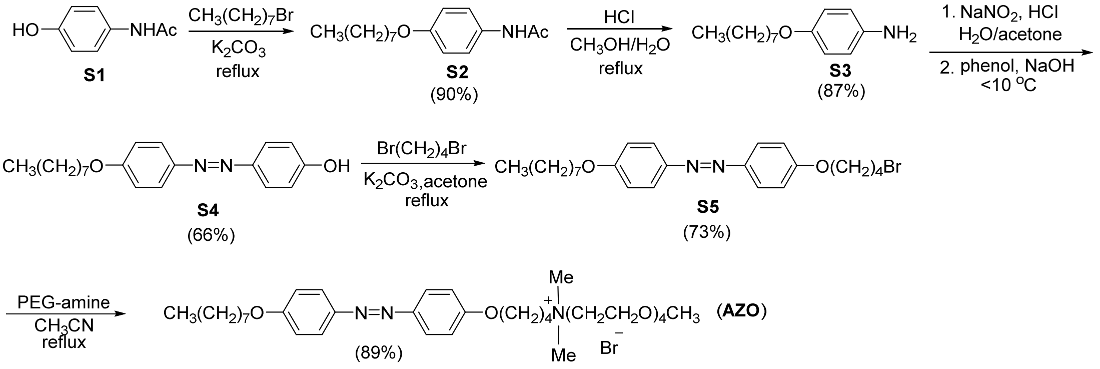

Scheme S2. Syntheses of AZO.[1]

***N,N*-dimethyl-*N*-(4-(4-((4-(octyloxy)phenyl)diazenyl)phenoxy)butyl)-2,5,8,11-tetraoxatridecan-13-aminium bromide (AZO):** to a solution of PEG-amine (102 mg, 0.44 mmol, 0.9 eq) in acetonitrile (15 mL) was added compound S5 (250 mg, 0.54 mmol, 1

eq). The mixture was stirred at 80 °C over 24h. The resulted mixture was cooled to r.t. and concentrated by removing the acetonitrile. The obtained solid was washed with ethyl acetate and CH_2Cl_2 to provide AZO (335 mg, 89% yield) as an orange solid. ^1H NMR (500 MHz, $CDCl_3$): δ 7.84-7.82 (m, 4 H), 6.98-6.95 (m, 4 H), 4.09 (t, J = 6.0 Hz, 2 H), 4.01-3.95 (m, 4 H), 3.88-3.87 (m, 2 H), 3.76-3.72 (m, 2 H), 3.64-3.62 (m, 2 H), 3.57-3.56 (m, 8 H), 3.50 -3.49 (m, 2 H), 3.38 (s, 6 H), 3.33 (s, 3 H), 2.03-1.97 (m, 2 H), 1.92-1.89 (m, 2 H), 1.81-1.76 (m, 2 H), 1.48-1.42 (m, 2 H), 1.35-1.23 (m, 8 H), 0.87 (t, J = 6.5 Hz, 3 H); ^{13}C NMR (125 MHz, $CDCl_3$): δ 161.2, 160.4, 147.1, 146.7, 124.3, 114.62, 114.60, 71.8, 70.4, 70.33, 70.30, 70.28, 70.1, 68.3, 67.0, 65.4, 64.9, 63.2, 58.9, 51.9, 31.7, 29.3, 29.14, 29.12, 25.93, 25.86, 22.6, 19.8, 14.0.

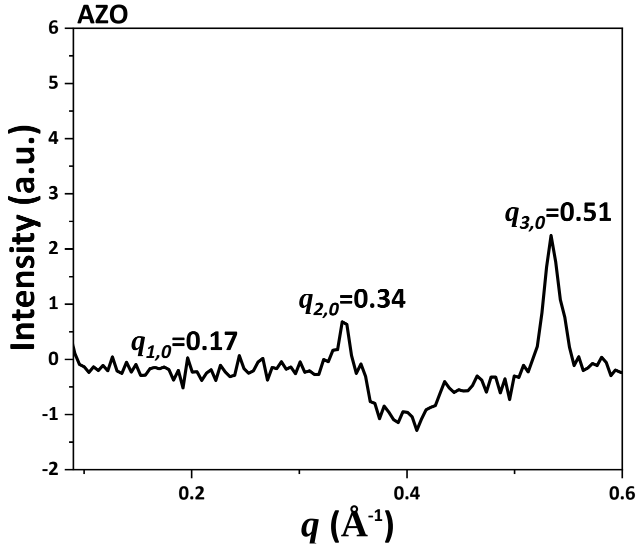

Figure S1. SAXS profile of AZO.

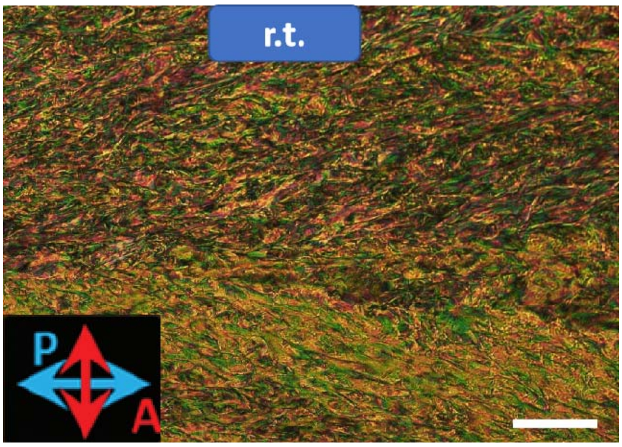

Figure S2. POM image of AZO at r.t.. Scale bar: 50 µm.

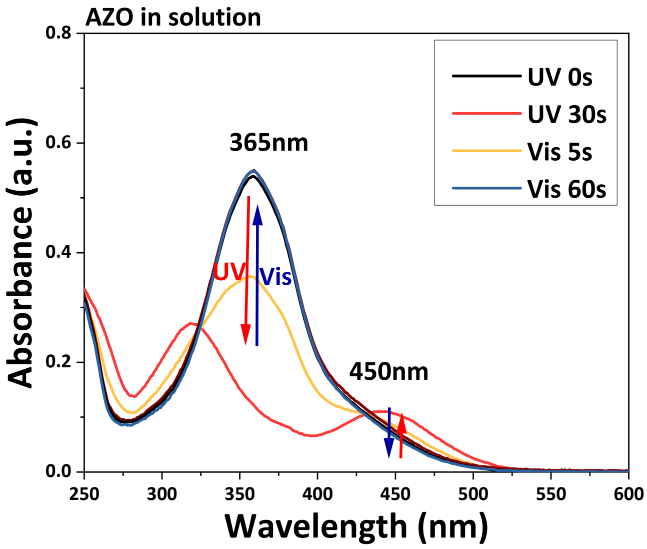

Figure S3. UV-Vis absorption changes of AZO in aqueous condition (9.6 μM) under UV/Vis irradiation at r.t..

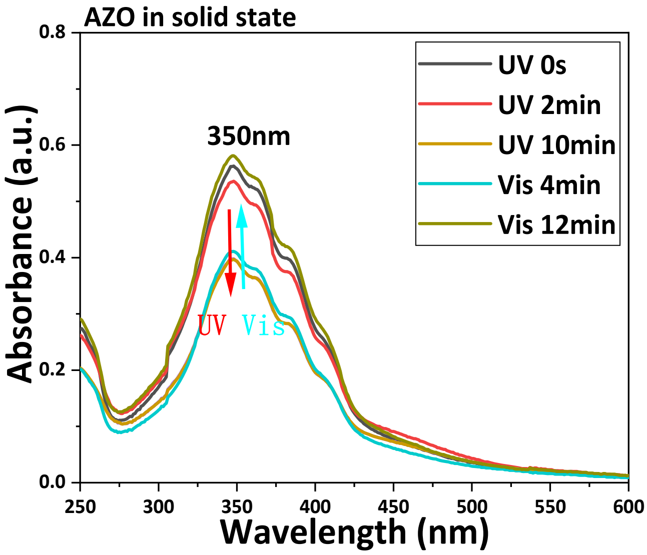

Figure S4. UV-Vis absorption changes of AZO in solid state under UV/Vis irradiation at r.t..

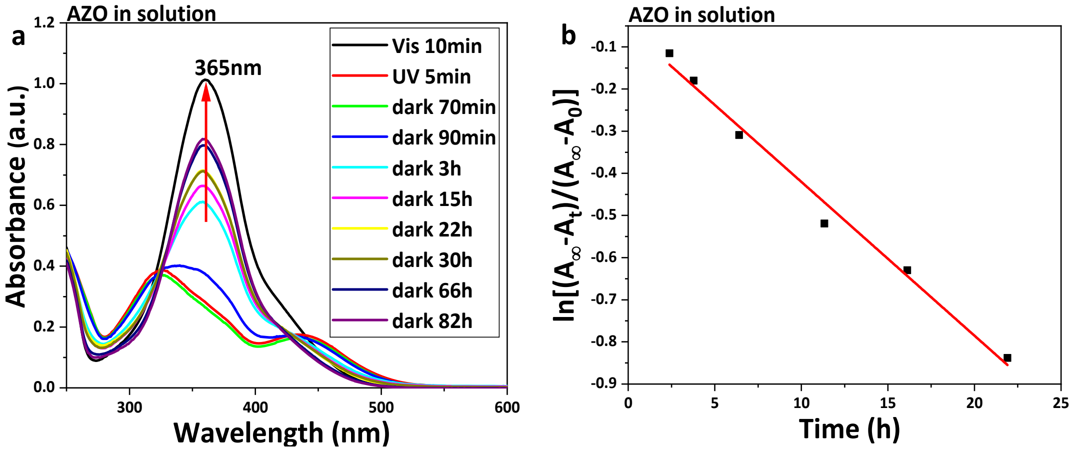

Figure S5. Temporal evolved UV-Vis absorption change of *cis*-AZO in aqueous solution (9.6 μM) in dark at r.t.. The π-π* absorption intensity at 365 nm is used for drawing the plotted graph. Equation of $\ln[(A_{\infty}-A_t)/(A_{\infty}-A_0)] = -\kappa_{rev}t$ is used for obtaining the thermodynamic *cis-to-trans* isomerization rate of *cis*-AZO in dark, affording κ_{rev} =0.0384 h^{-1}, and $t_{1/2} = \ln2/\kappa_{rev}$ is used for obtaining the half-life of *cis*-AZO in solution, affording $t_{1/2} = \ln2/0.0384 = 18.0$ h. A_{∞} is the absorption intensity of *trans*-AZO rich state after *cis-to-trans* isomerization. A_t is the absorption intensity of AZO at "t" time. A_0 is the absorption intensity of *cis*-AZO rich state after UV irradiation.

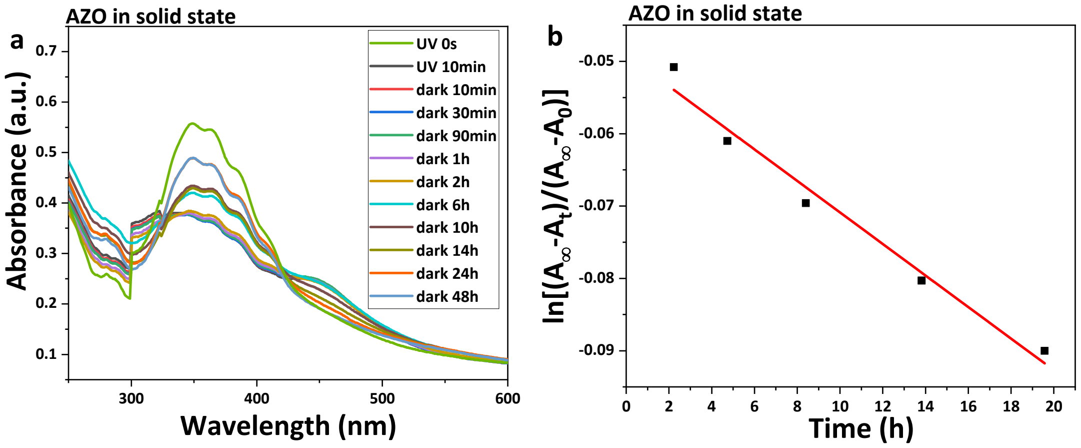

Figure S6. Temporal evolved UV-Vis absorption change of solid-state *cis*-AZO in dark at r.t.. Similar as that in solution, with equations of $\ln[(A_{\infty}-A_t)/(A_{\infty}-A_0)] = -\kappa_{rev}t$ and $t_{1/2} = \ln2/\kappa_{rev}$, the half-life (14.4 h) of *cis*-AZO in solid state could be obtained.

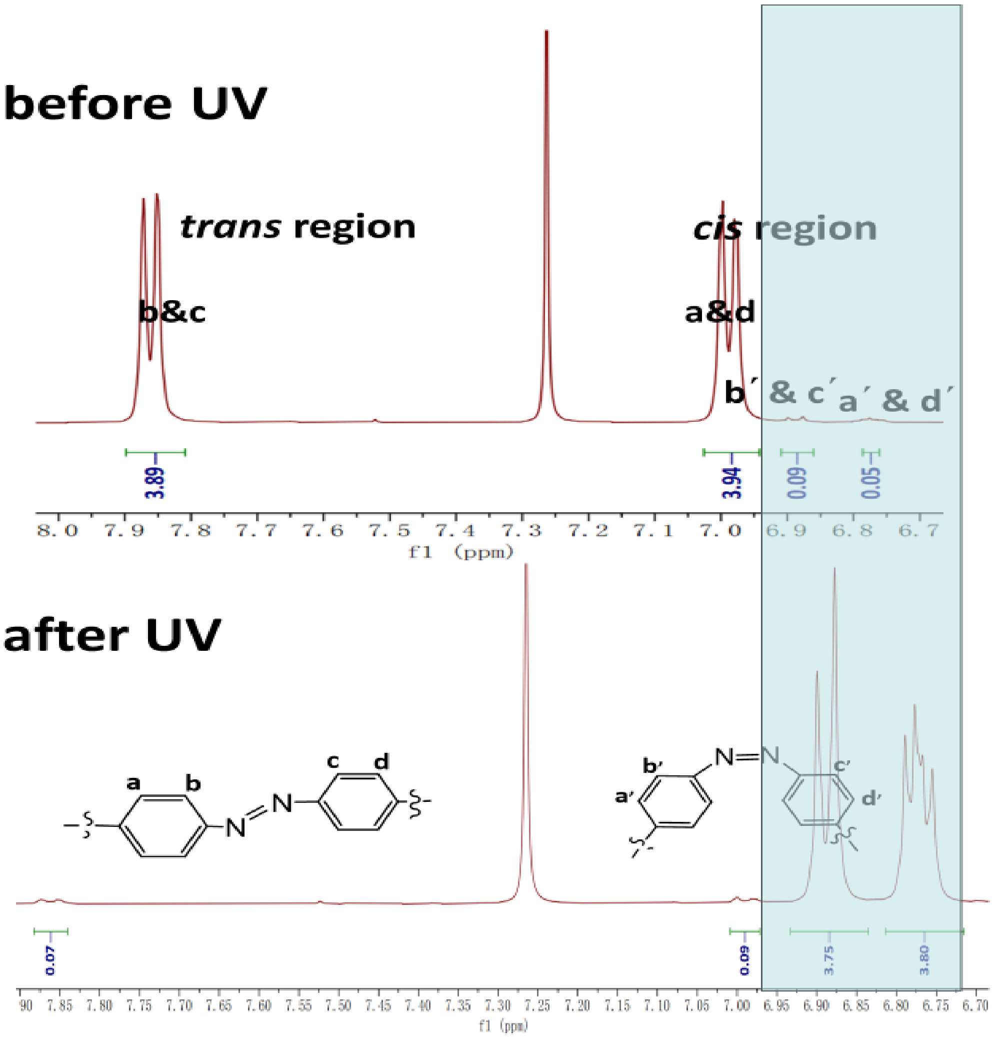

Figure S7. ^{1}H NMR investigation of *trans-to-cis* isomerization of AZO in $CDCl_3$ under UV irradiation. *Cis*-AZO% = 1.8% and 94% could be deduced from the ^{1}H NMR signal assignments for *trans*- and *cis*-AZO in $CDCl_3$ before and after UV irradiation.

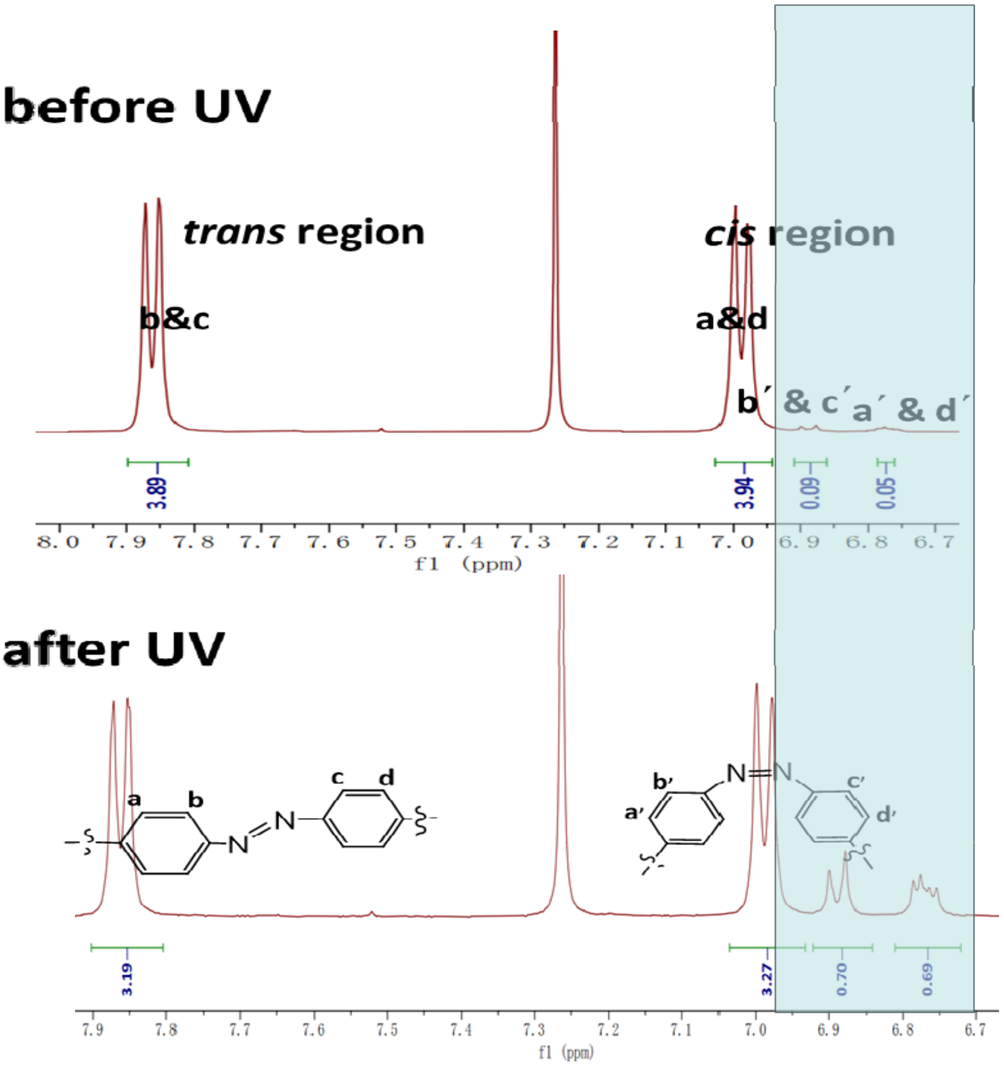

Figure S8. ^{1}H NMR investigation of *trans-to-cis* isomerization of solid-state AZO under UV irradiation. *Cis*-AZO% = 1.8% and 18% could be deduced from the ^{1}H NMR signal assignments for *trans*- and *cis*-AZO in solid state before and after UV irradiation.

3. Fabrication of Alg-AZO and Alg fibers

Alg-AZO fibers with different contents of Ca^{2+}: such fibers were prepared by following a similar synthetic procedure of Alg-AZO but injecting the solution mixture of Alg and AZO (charge ratio = 20:1) into $CaCl_2$ solutions (50 mL) with different mass concentrations.

Alg-AZO fibers with different charge ratios of Alg:AZO: such fibers were prepared by following a similar synthetic procedure of Alg-AZO but injecting the solution mixture of Alg and AZO with different charge ratios into a $CaCl_2$ solution (0.6%, 50 mL).

Alg fibers: this fiber was prepared by following a similar synthetic procedure of Alg-AZO but without using AZO.

4. Characterizations of Alg-AZO and Alg fibers

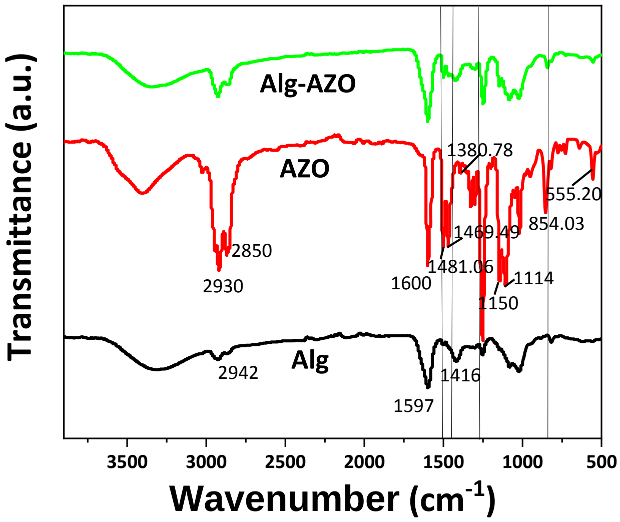

Figure S9. FT-IR profiles of Alg, AZO and Alg-AZO. FT-IR spectrum of Alg shows peaks at 3278 cm^{-1} from O-H stretching vibration, 2942cm^{-1} from C-H stretching vibration, 1597 and 1416 cm^{-1} from asymmetric and symmetric stretching vibrations of -COO-, 1027 cm^{-1} from antisymmetric stretching vibration of C-O.[2] FT-IR spectrum of AZO shows peaks at 2930 and 2850 cm^{-1} from antisymmetric and symmetric stretching vibrations of - CH_2-, 1600 cm^{-1} from benzene backbone vibration,[3] 1481.06, 1469.49, 1380.78 and 1114 cm^{-1} from C-H stretching vibration of quaternary ammonium head,[4] 1150 cm^{-1} from stretching vibration of C-O-C.[5] FT-IR spectrum of Alg-AZO fiber shows peaks at 3421 cm^{-1} from O-H stretching vibration, 2925 cm^{-1} from C-H stretching vibration, 1597 and 1416 cm^{-1} from asymmetric and symmetric stretching vibrations of -COO-, 1499.12 cm^{-1}, 1422.56 cm^{-1} and 1470 cm^{-1} from C-H stretching vibration of quaternary ammonium head, 1243 cm^{-1} from C-O-C bond stretching vibration, 1027 cm^{-1} from antisymmetric stretching vibration of C-O. These peaks indicate the successful complexation between Alg and AZO.

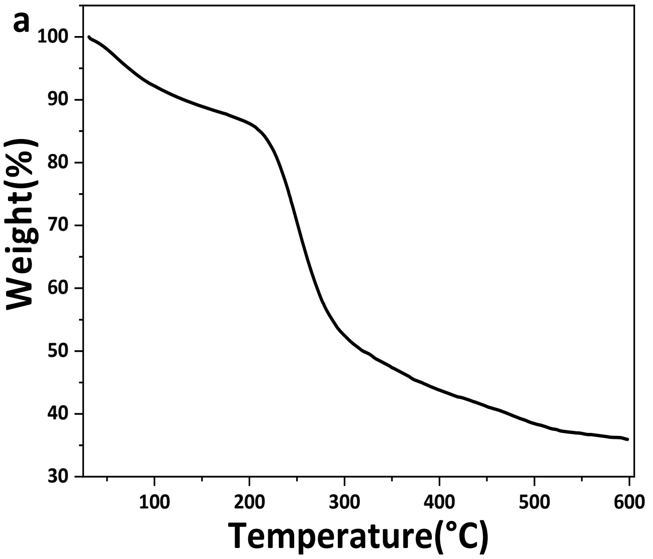

Figure S10. TGA profile of Alg-AZO.

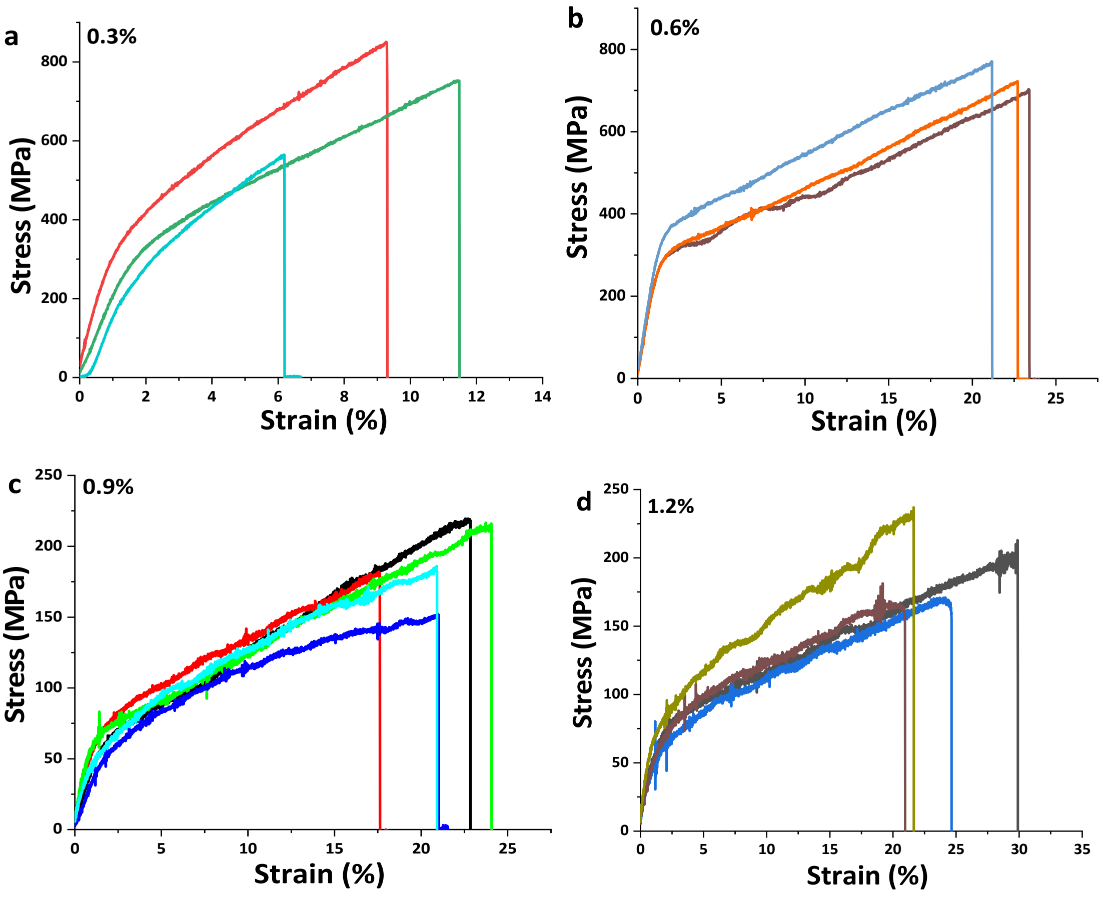

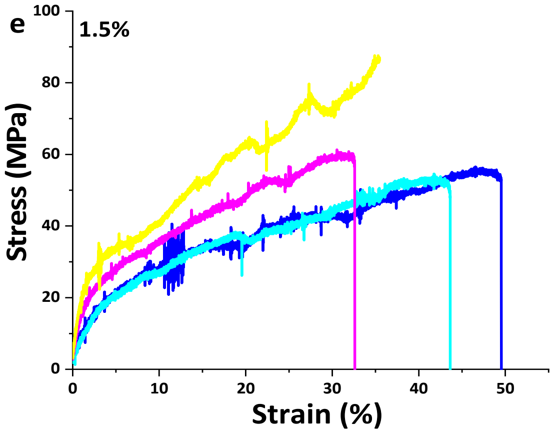

Figure S11. Stress-strain profiles of Alg-AZO fibers extruded from $CaCl_2$ solutions in mass concentrations of a) 0.3%, b) 0.6%, c) 0.9%, d) 1.2% and e)1.5%. For all the fibers, Alg : AZO = 20:1.

Table S1. Mechanical properties of Alg-AZO fibers extruded from $CaCl_2$ solutions in different mass concentrations. For all the fibers, Alg : AZO = 20:1.

Entry	$CaCl_2$%	Tensile strength ± S.D. (MPa)	Young's modulus ± S.D. (GPa)	Strain ± S.D. (%)	Toughness ± S.D. (MJ/m^3)
1	0.3	722.55 ± 145.06	21.06 ± 5.37	11.65 ±2.48	43.41 ± 19.52
2	0.6	731.80 ± 35.50	12.16 ± 3.78	22.40 ± 1.13	111.88 ± 2.96
3	0.9	205.10 ± 29.83	4.73 ± 1.31	21.26± 2.45	28.70 ± 5.29
4	1.2	187.46 ± 38.17	5.36 ± 1.38	21.30 ± 4.60	27.77 ± 7.58
5	1.5	62.35 ± 7.52	1.94 ± 0.56	41.67± 8.61	17.54 ± 2.75

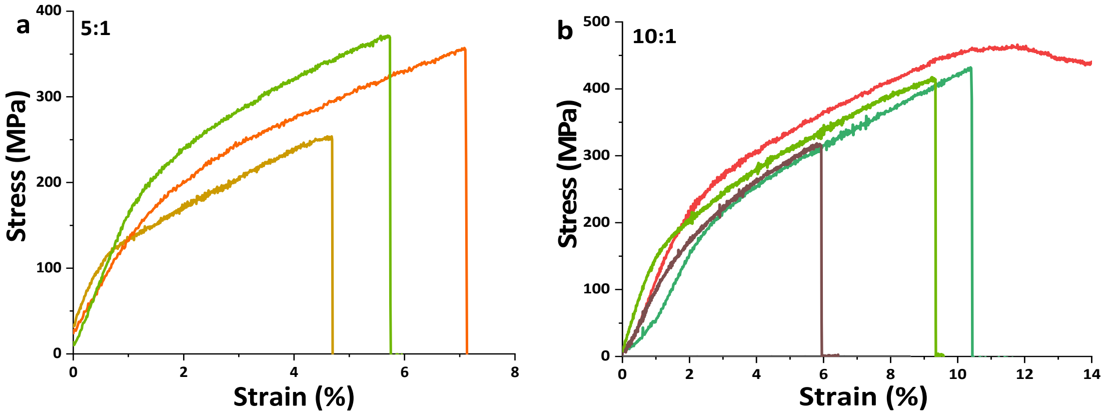

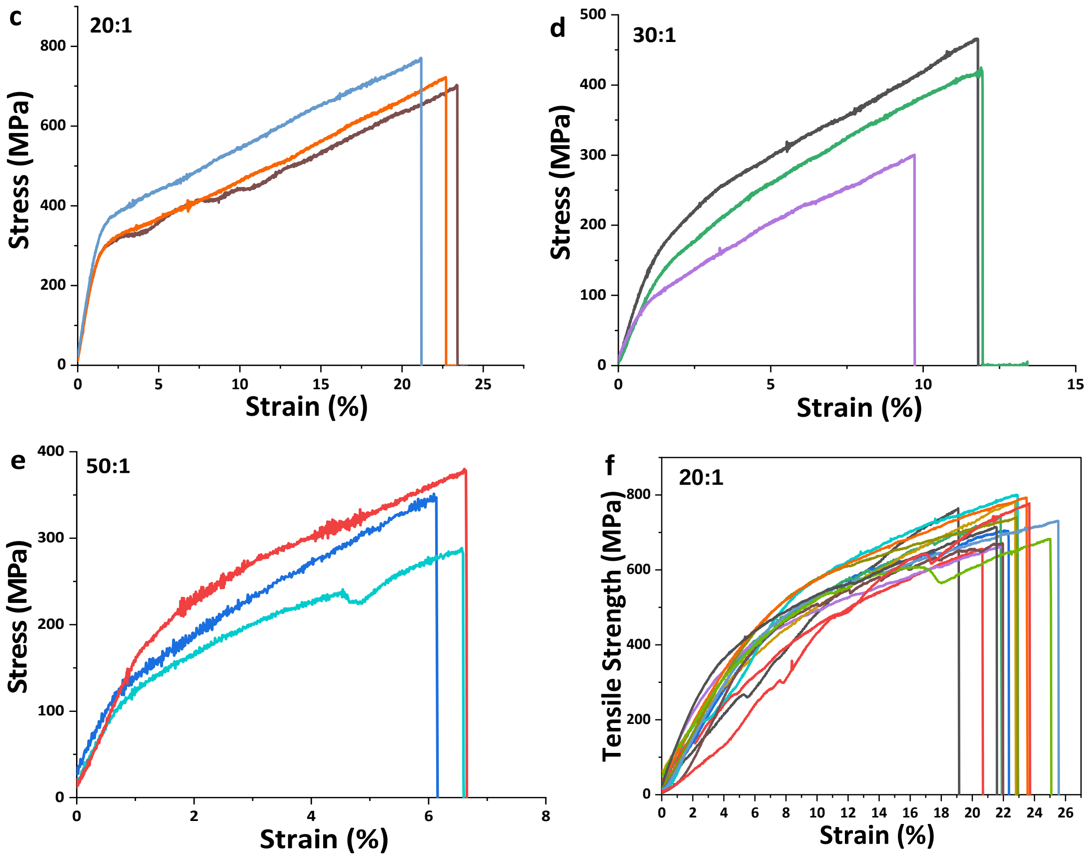

Figure S12. Stress-strain profiles of Alg-AZO fibers fabricated with stoichiometric charge ratios of Alg:AZO in a) 5:1, b) 10:1, c) 20:1, d) 30:1, e) 50:1 and f) 20:1 for 15 times of repeating tests. All the fibers were extruded from 0.6% $CaCl_2$ solution.

Table S2. Mechanical properties of Alg-AZO fibers fabricated with different stoichiometric charge ratios of Alg:AZO. All the fibers were extruded from 0.6% $CaCl_2$ solution.

Entry	Alg:AZO	Tensile strength ± S.D. (MPa)	Young's modulus ± S.D. (GPa)	Strain ± S.D. (%)	Toughness ± S.D. (MJ/m^3)
1	5:1	327.38 ±64.06	16.04 ± 2.21	5.81 ±1.22	15.46± 1.50
2	10:1	393.68 ± 54.56	10.92± 2.90	8.61 ± 2.16	22.59 ± 7.76
3	20:1	731.80 ± 35.50	12.16 ± 3.78	22.40±1.13	111.88 ± 2.96
4	30:1	396.91 ± 85.90	13.91±3.11	11.13± 1.23	28.84± 9.29
5	50:1	338.56 ± 41.26	16.94 ± 5.41	6.39 ± 2.12	14.45 ± 2.37

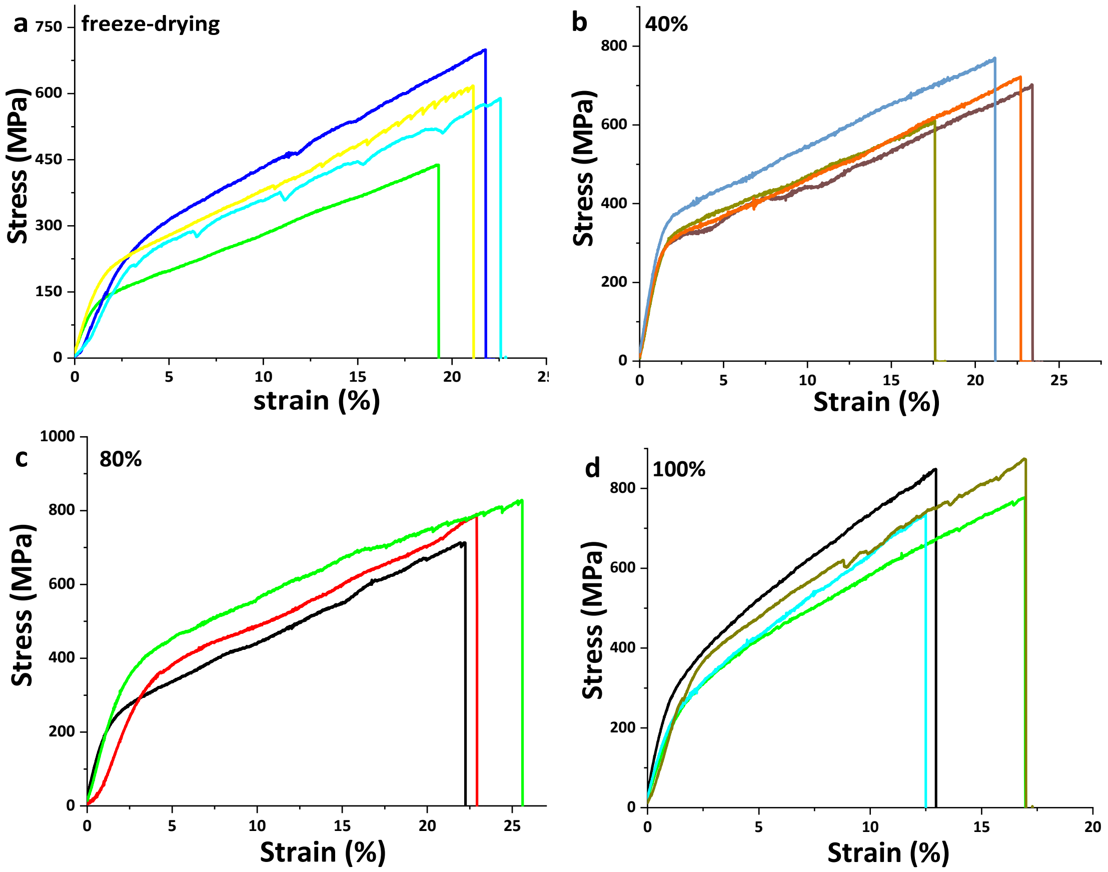

Figure 13. Stress-strain profiles of Alg-AZO fibers incubated in a) freeze-drying, b) RH = 40%, c) RH = 50%, and d) RH = 100%. All the fibers were fabricated with stoichiometric charge ratio of Alg : AZO = 20:1 and extruded from 0.6% $CaCl_2$ solution.

Table S3. Mechanical properties of Alg-AZO fibers incubated in different RHs. All the fibers were fabricated with stoichiometric charge ratio of Alg : AZO = 20:1 and extruded from 0.6% $CaCl_2$ solution.

Entry	RH (%)	Tensile strength ± S.D. (MPa)	Young's modulus ± S.D. (GPa)	Strain ± S.D. (%)	Toughness ± S.D. (MJ/m^3)
1	freeze-drying	634.35±56.75	14.77 ±1.00	21.12±0.11	86.32 ±7.21
2	40	731.80 ± 35.50	12.16 ± 3.78	22.40±1.13	111.88 ± 2.96
3	80	775.21±59.96	17.52±0.99	23.52±1.70	112.23 ±9.88
4	100	821.29±59.89	14.42 ±0.59	14.09±2.34	85.68 ±12.61

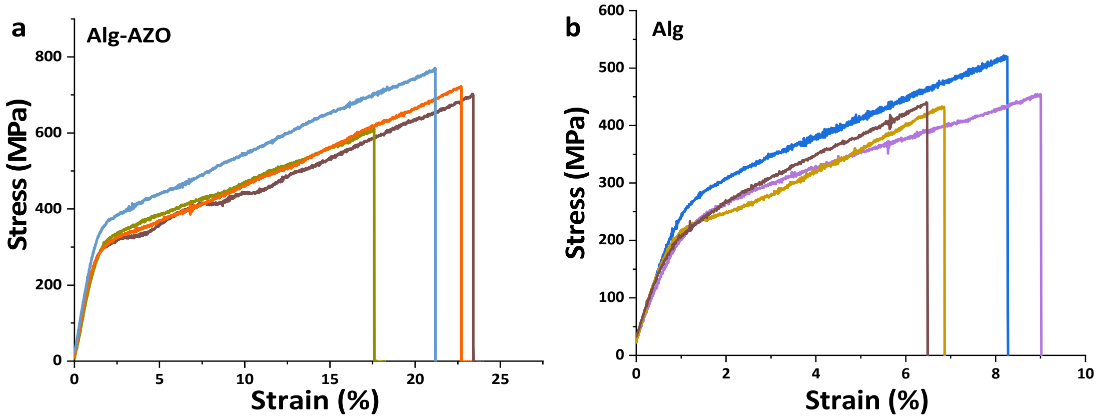

Figure S14. Stress-strain profiles of Alg-AZO and Alg fibers.

Table S4. Mechanical properties of Alg-AZO and Alg fibers.

Entry	Fiber	Tensile strength ± S.D. (MPa)	Young's modulus ± S.D. (GPa)	Strain ± S.D. (%)	Toughness ± S.D. (MJ/m^3)
1	Alg-AZO	731.80 ± 35.50	12.16 ± 3.78	22.40 ± 1.13	111.88 ± 2.96
2	Alg	461.65 ± 41.26	23.63±2.14	7.58 ± 1.18	24.2 ± 6.02

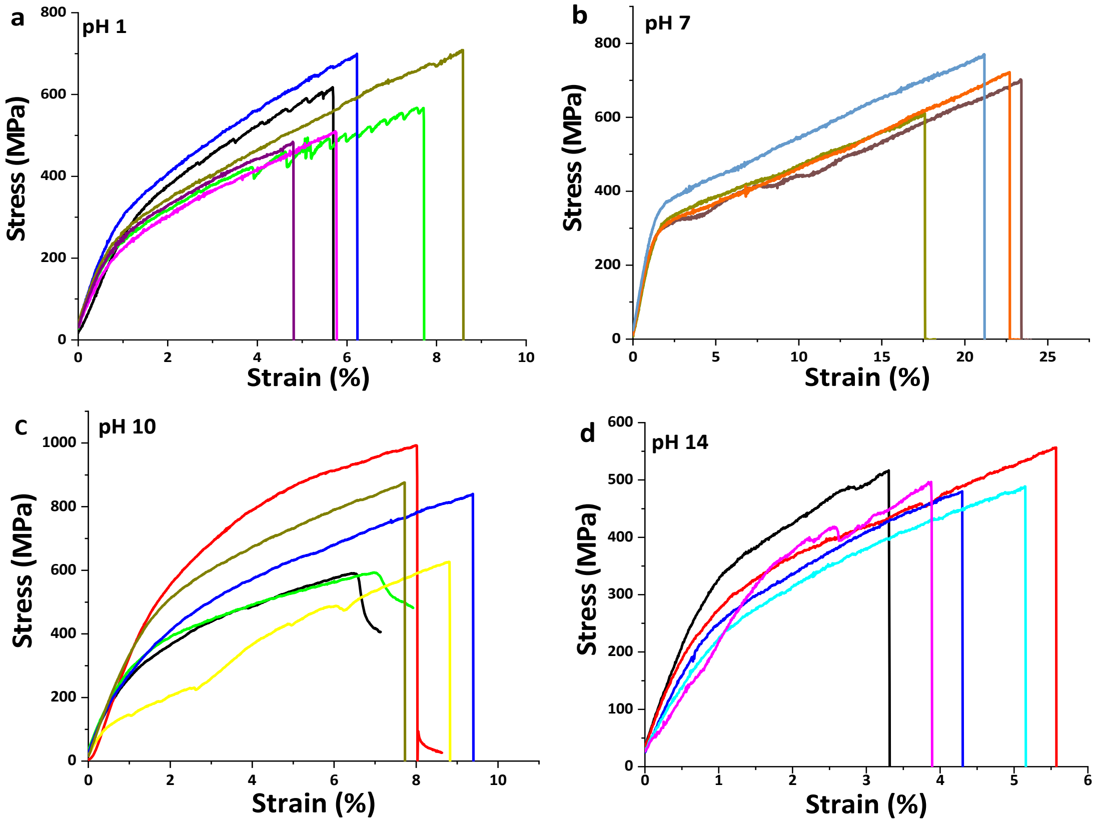

Figure 15. Stress-strain profiles of Alg-AZO fibers treated by a) pH 1, b) pH 7, c) pH 10 and d) pH 14.

Table S5. Mechanical properties of Alg-AZO fibers treated by different pH conditions.

Entry	pH	Tensile strength ± S.D.(MPa)	Young's modulus ± S.D. (GPa)	Strain ± S.D. (%)	Toughness ± S.D. (MJ/m^3)
1	1	724.77± 77.77	26.39 ± 2.42	10.10 ± 0.8	47.23 ± 8.86
2	7	731.80 ± 35.50	12.16 ± 3.78	22.40 ± 1.13	111.88 ± 2.96
3	10	600.73 ± 22.56	19.11 ± 1.29	7.19 ± 1.10	32.56 ± 2.62
4	14	488.48 ± 8.18	27.51 ± 3.33	4.70 ± 0.70	13.81 ± 2.45

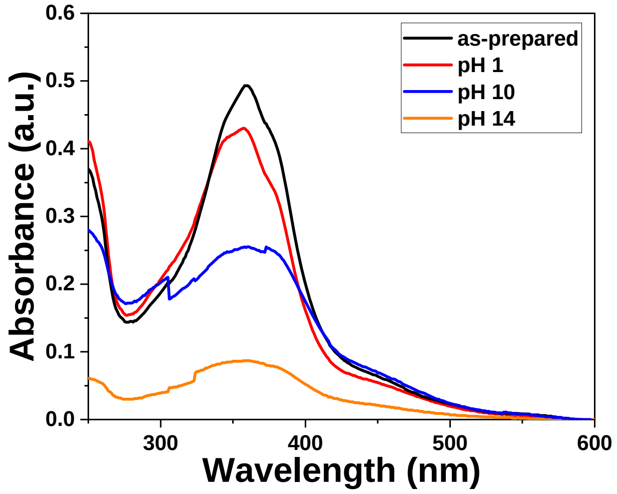

Figure S16. UV-Vis absorption spectra of Alg-AZO fibers treated by a) pH 1, b) pH 10 and c) pH 14.

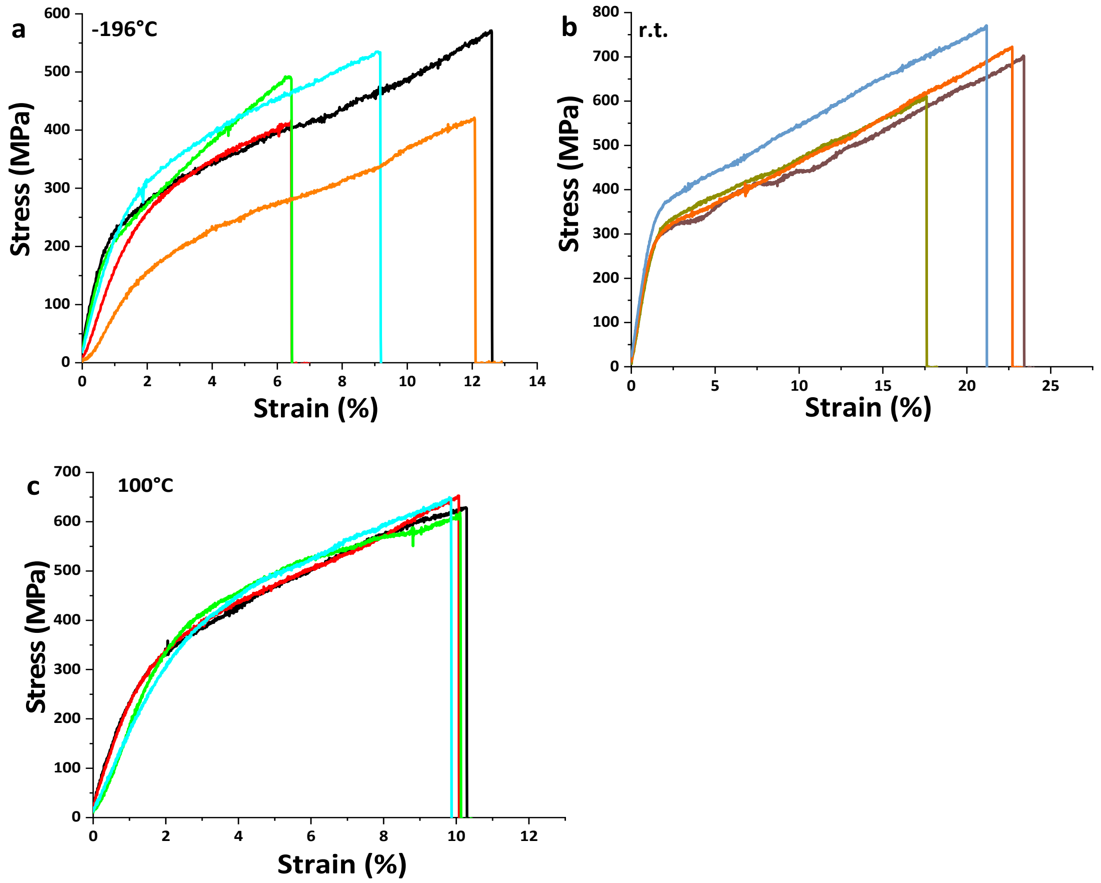

Figure S17. Stress-strain profiles of Alg-AZO fibers treated by a) liquid nitrogen, b) r.t. and c) 100 °C.

Table S6. Mechanical properties of Alg-AZO fibers treated by different temperatures.

Entry	Temperature (°C)	Tensile strength ± S.D.(MPa)	Young's modulus ± S.D. (GPa)	Strain ± S.D. (%)	Toughness ± S.D. (MJ/m^3)
1	-196	481.43 ± 59.94	26.50 ± 0.23	7.28 ± 1.56	24.72 ± 9.07
2	r.t.	731.80 ± 35.50	12.16 ± 3.78	22.40 ± 1.13	111.88 ± 2.96
3	100	636.75 ±16.29	16.70± 0.63	10.06 ± 0.19	44.73± 0.85

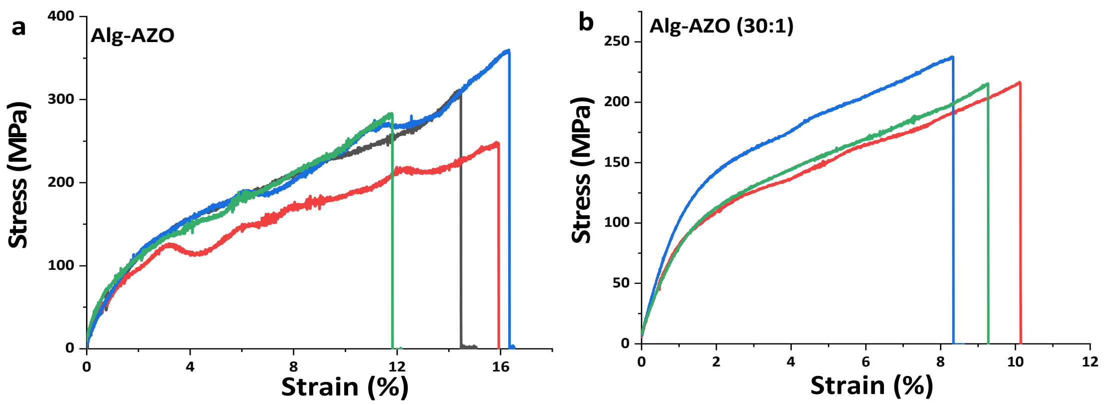

Figure S18. Stress-strain profiles of a) Alg-AZO and b) Alg-AZO (30:1) fibers after UV irradiation for 5min at r.t..

Table S7. Mechanical properties of Alg-AZO and Alg-AZO (30:1) fibers after UV irradiation for 5min at r.t..

**Entry**	**Fiber**	**Tensile strength ± S.D.(MPa)**	**Young's modulus ± S.D. (GPa)**	**Strain ± S.D. (%)**	**Toughness ± S.D. (MJ/m^3)**
1	Alg-AZO	299.08 ± 47.09	7.73± 0.75	14.59 ± 2.04	26.95 ± 5.66
2	Alg-AZO (30:1)	196.68 ± 53.45	8.41 ± 1.00	9.21 ± 0.90	14.08 ± 0.67

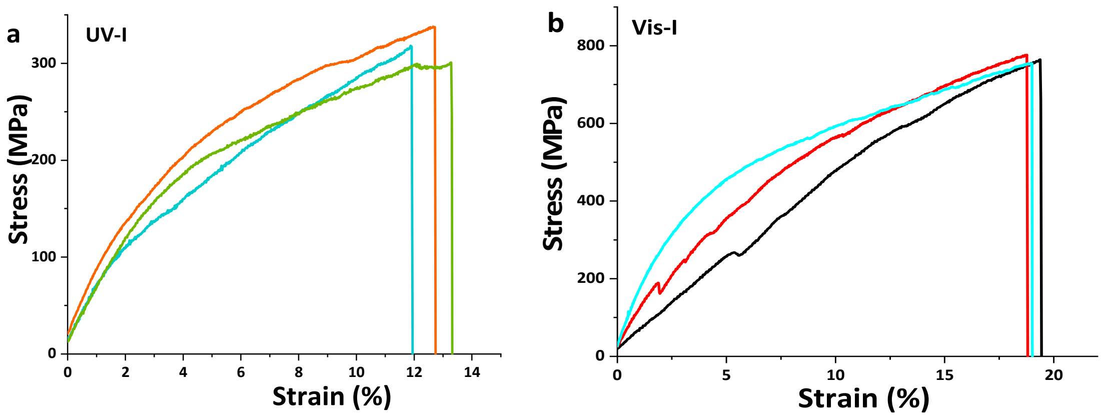

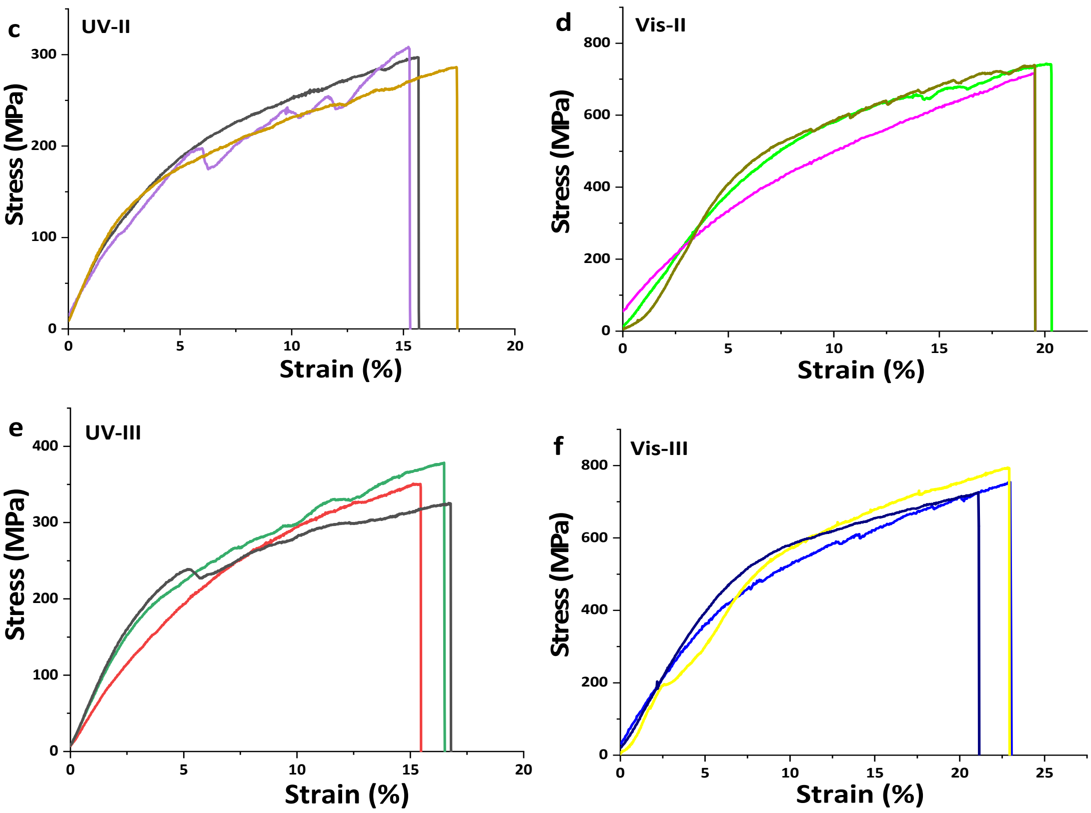

Figure S19. Stress-strain profiles of Alg-AZO fibers in three repeating UV-Vis irradiation tests at r.t.. UV and Vis irradiation times: 5min.

Table S8. Mechanical properties of Alg-AZO fibers in three repeating UV-Vis irradiation tests at r.t..

Entry	treatments	Tensile strength ± S.D.(MPa)	Young's modulus ± S.D. (GPa)	Strain ± S.D. (%)	Toughness ± S.D. (MJ/m^3)
1	UV-I	319.30 ± 17.25	7.31±0.76	12.63 ± 0.68	26.55±3.24
2	Vis-I	764.96± 11.24	13.48 ± 1.72	19.03± 0.29	92.42± 8.58
3	UV-II	296.52 ± 9.87	9.11± 0.83	16.10 ± 1.10	32.23± 2.90
4	Vis-II	724.34± 15.53	13.85± 1.26	20.03± 0.87	97.93± 11.9
5	UV-III	359.62 ±30.04	10.47± 0.42	16.22±0.72	39.57 ± 3.57
6	Vis-III	775.34 ± 30.39	11.8±0.77	21.98 ±1.46	112.67± 8.69

5. Intermolecular interaction analyses by computational calculation

The geometric optimizations of the ground state of free molecules were performed within Gaussian 09 program package (Revision D.01)[6] at the level of density

functional theory (DFT) B3LYP functional[7] coupled with Grimme D3 dispersion correction[8] and 6-31G(d,P) basis set. Meanwhile, an assumption that there would be H-bond interaction between –OH group of polysaccharide chain and oxygen atom of PEG was applied in the modeling process, with considering the hydrophilic property of PEG chain and the hydrophobic property of azobenzene-containing chain. To confirm the nature of obtained minima, vibrational frequency calculations were then carried out at the same level of theory as geometric optimizations. To analyze the weak interactions among molecules, the improvement version of independent gradient model based on Hirshfeld partition of molecular density (IGMH) method was used.[9, 10] As a quantitative descriptor of inter-fragment interaction, δ_g^{inter} indices between Alg and *trans-/cis*-AZO were also calculated, which were 0.76 a.u. for Alg & *trans*-AZO and 1.18 a.u. for Alg & *cis*-AZO, respectively.

6. Biological studies of Alg-AZO fiber

Cell toxicity of Alg-AZO

The cell toxicity was assessed according to the reported literature with some modifications.[11] Mouse fibroblast cells (L929) were seeded to a 96-well plate (10000 cells per well) and cultured in DMEM medium, supplemented with 10% FBS and 1% penicillin-streptomycin in an incubator containing 5% CO_2 at 37 °C for 24 h. The aqueous suspensions with different mass concentrations of Alg-AZO (62.50, 31.25, 15.63, 7.82, 3.91 μg/mL) were prepared by adding the short pieces of fiber material into aqueous solutions and further incubated for 24 h. Then, CCK-8 solution (100 μL, 10%) was added and incubated for another 1 h at 37 °C. After removing 90 μL of supernatant to another 96-well plate, the absorbance at 450 nm was determined by a microplate reader to calculate the cell viability according to the formula:

Cell viability = $(A_e - A_o)/ (A_c - A_o) \times 100\%$ (1)

where, A_o, A_e and A_c represent the absorbance of blank group, experimental group and control group, respectively.

Fluorescence staining of cells (live/dead)

For fluorescence (live/dead) measurement, a mixture of Calcein-AM (1 μL), PI (1 μL) and PBS (1000 μL) was prepared for cells staining. Mouse fibroblast cells (L929) were seeded to a 6-well plate (100000 cells per well) and cultured in DMEM medium, supplemented with 10% FBS and 1% penicillin-streptomycin in an incubator

containing 5% CO_2 at 37 °C for 24 h. The aqueous suspensions of Alg-AZO with different mass concentrations of 62.50, 31.25, 15.63, 7.82, 3.91 μg/mL were added and further incubated for 24 h. Then the suspensions of Alg-AZO were removed and the DMEM medium was washed twice with PBS solution. The staining solution (1 mL) was added and incubated at 37 °C for 30 min, and the images were recorded by a confocal laser scanning microscopy (CLSM, Leica, SP8, Germany). Green fluorescence excited from Calcein-AM labels living cells, red fluorescence excited from PI labels dead cells and an overall image of the red fluorescence and green is also merged.

References

[1] L. Zhang, Z. Tang, L. Hou, Y. Qu, Y. Deng, C. Zhang, C. Xie, Z. Wu, *Analyst* **2020**, *145*, 1641-1645.

[2] M. Fertah, A. Belfkira, E. m. Dahmane, M. Taourirte, F. Brouillette, *Arabian J. Chem.* **2017**, *10*, S3707-S3714.

[3] R. Yang, D. Zhao, G. Dong, Y. Liu, D. Wang, in *Crystals, Vol. 8*, **2018**.

[4] L. Wei, J. Zhang, F. Luan, W. Tan, Q. Li, F. Dong, Z. Guo, *Starch - Stärke* **2018**, *70*, 1700266.

[5] C.-F. Chen, S.-H. Chen, R.-F. Chen, K.-F. Liu, Y.-R. Kuo, C.-K. Wang, T.-M. Lee, Y.-H. Wang, in *International Journal of Molecular Sciences, Vol. 24*, **2023**.

[6] M. J. Frisch, G. W. Trucks, H. B. Schlegel, G. E. Scuseria, M. A. Robb, J. R. Cheeseman, G. Scalmani, V. Barone, B. Mennucci, G. A. Petersson, H. Nakatsuji, M. Caricato, X. Li, H. P. Hratchian, A. F. Izmaylov, J. Bloino, G. Zheng, J. L. Sonnenberg, M. Hada, M. Ehara, K. Toyota, R. Fukuda, J. Hasegawa, M. Ishida, T. Nakajima, Y. Honda, O. Kitao, H. Nakai, T. Vreven, J. A. Montgomery, J. E. Peralta, F. Ogliaro, M. Bearpark, J. J. Heyd, E. Brothers, K. N. Kudin, V. N. Staroverov, T. Keith, R. Kobayashi, J. Normand, K. Raghavachari, A. Rendell, J. C. Burant, S. S. Iyengar, J. Tomasi, M. Cossi, N. Rega, M. J. Millam, M. Klene, J. E. Knox, J. B. Cross, V. Bakken, C. Adamo, J. Jaramillo, R. Gomperts, R. E. Stratmann, O. Yazyev, A. J. Austin, R. Cammi, C. Pomelli, J. W. Ochterski, R. L. Martin, K. Morokuma, V. G. Zakrzewski, G. A. Voth, P. Salvador, J. J. Dannenberg, S. Dapprich, A. D. Daniels, Ö. Farkas, J. B. Foresman, J. V. Ortiz, J. Cioslowski, D. J. Fox, *09 Gaussian, Revision D.01, Gaussian, Inc., Wallingford, CT* **2013**.

[7] A. D. Becke, *J. Chem. Phys.* **1993**, *98*, 5648-5652.

[8] S. Grimme, S. Ehrlich, L. Goerigk, *J. Comput. Chem.* **2011**, *32*, 1456-1465.

[9] T. Lu, Q. Chen, *J. Comput. Chem.* **2022**, *43*, 539-555.

[10] T. Lu, F. Chen, *J. Comput. Chem.* **2012**, *33*, 580-592.

[11] S. Zhao, Y. Xia, Q. Lan, Q. Wu, X. Feng, Y. Liu, *ACS Appl. Nano Mater.* **2023**, *6*, 8643-8654.
